# Influence of Ligand and Nuclearity on the Cytotoxicity of Cyclometallated C^N^C Platinum(II) Complexes

**DOI:** 10.1002/chem.202002517

**Published:** 2020-10-15

**Authors:** Angélique Kergreis, Rianne M. Lord, Sarah J. Pike

**Affiliations:** ^1^ School of Chemistry and Biosciences Faculty of Life Sciences University of Bradford Bradford, West Yorkshire BD7 1DP UK; ^2^ School of Chemistry University of East Anglia Norwich Research Park Norwich NR4 7TJ UK; ^3^ School of Chemistry University of Birmingham Edgbaston Birmingham B15 2TT UK

**Keywords:** anticancer, bioinorganic chemistry, cyclometallated platinum(II), multinuclear complexes, phosphine ligands

## Abstract

A series of cyclometallated mono‐ and di‐nuclear platinum(II) complexes and the parent organic ligand, 2,6‐diphenylpyridine **1** (HC^N^CH), have been synthesized and characterized. This library of compounds includes [(C^N^C)Pt^II^(**L**)] (**L**=dimethylsulfoxide (DMSO) **2** and triphenylphosphine (PPh_3_) **3**) and [((C^N^C)Pt^II^)_2_(**L‘**)] (where **L‘**=*N*‐heterocycles (pyrazine (pyr) **4**, 4,4‘‐bipyridine (4,4‘‐bipy) **5** or diphosphine (1,4‐bis(diphenylphosphino)butane (dppb) **6**). Their cytotoxicity was assessed against four cancerous cell lines and one normal cell line, with results highlighting significantly increased antiproliferative activity for the dinuclear complexes (**4**–**6**), when compared to the mononucleated species (**2** and **3**). Complex **6** is the most promising candidate, displaying very high selectivity towards cancerous cells, with selectivity index (SI) values >29.5 (A2780) and >11.2 (A2780cisR), and outperforming cisplatin by >4‐fold and >18‐fold, respectively.

## Graphical abstract

**A study of the cytotoxicity** of a library of cyclometallated mono‐ and di‐nuclear platinum(II)‐complexes, against a range of ovarian and breast cancer cell lines, identifies the importance of both ligand type and nuclearity on their antiproliferative activity. The lead candidate, a di‐nuclear platinum(II)‐phosphine complex, exhibits very high selectivity towards cancerous cells, with a selectivity index (SI) value >11.2 against cisplatin‐resistant ovarian cancer cells and outperforming monodentate analogues by >11‐fold and cisplatin by >18‐fold.

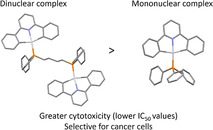

## Introduction

Since the approval of the chemotherapeutic drug cisplatin, by the FDA in 1978, platinum(II)‐based complexes have become integral in the clinical treatment of a range of different cancers.^[1]^ However, clinical platinum(II) anticancer drugs have several drawbacks associated with their use, including intrinsic and acquired resistance, lack of selectivity and neurotoxic side effects.^[2]^ Consequently, this has generated great interest in the development of alternative platinum(II) complexes which have the potential to address the significant disadvantages linked to current clinical platinum complexes.[^3^, ^4^]

Cyclometallated platinum(II) complexes have emerged as attractive alternatives to existing clinical antiproliferative platinum drugs, and several compounds were reported to possess moderate to potent cytotoxicity against cancer cell lines that are resistant to current platinum(II) anticancer drugs.[^5^, ^6^] To date, cyclometallated platinum(II) compounds bearing a diverse range of tridentate organic π‐ligands scaffolds including, C^N^S,^[8]^ C^N^N,^[9]^ N^C^N^[10]^ and N^N^N^[11]^ have been reported to display up to sub‐micromolar potency against a range of human cell lines for example, breast adenocarcinoma (MCF‐7) and colon carcinoma (HCT116).^[5]^

Early reports from Lowe and co‐workers outlined the potent cytotoxicity of cyclometallated platinum(II)‐complexes incorporating a N^N^N terpyridine (terpy) ligand scaffold, and a range of *N*‐heterocycles and thiolates in the fourth coordination site, against a panel of human cancer cell lines including two human ovarian carcinomas, A2780 and A2780cisR.^[12]^ Following on from this initial work, several groups have demonstrated the significant antiproliferative properties of platinum(II)‐terpy systems, with reports of the biological activity rivalling that of cisplatin in a diverse array of human cancer cell lines.[^13^, ^14^, ^15^, ^16^, ^17^, ^18^] However, the cytotoxicity of cyclometallated, C^N^C platinum(II) compounds has not been widely studied.[^19^, ^20^] Klein and co‐workers showed that a series of cyclometallated complexes based on [(C^N^C)Pt^II^(**L**)], wherein C^N^C is a tridentate dianionic cyclometallating motif bearing a range of aryl groups; including phenyl, naphthyl and dibenzoacridine derivatives and **L**=DMSO or acetonitrile (MeCN), displayed good to moderate cytotoxicity against human cell lines; colorectal adenocarcinoma (HT‐29) and breast adenocarcinoma (MDA‐MB‐231).^[21]^

Polynuclear platinum(II) complexes represent an important growing class of anticancer agents with potential clinical significance.[^22^, ^23^, ^24^, ^25^] Developing an understanding of the structure–activity relationships (SARs) within classes of biologically active complexes is integral for optimization of their performance.[^12^, ^26^, ^27^, ^28^, ^29^] Che and co‐workers probed the importance of nuclearity on the SAR of a series of cyclometallated, tridentate C^N^N platinum(II) complexes, establishing that dinuclear species display more than one order of magnitude higher cytotoxicity than their monomeric analogues against five human carcinoma cell lines.[^15^, ^30^] Che and co‐workers also established the significance of linker size on the antiproliferative activity of a related series of [((C^N^N)Pt)_2_(*μ*‐NHC)]^+^ platinum(II) complexes where HC^N^N=6‐phenyl‐2,2‘‐bipyridyl and *μ*‐NHC=a bridging *N*‐heterocyclic carbene ligand. They showed that through increasing the length of the linker between the two metal centres, up to a 2‐fold increase in cytotoxicity was induced against a panel of human cell lines, including cervix epithelioid adenocarcinoma (HeLa), liver hepatocellular carcinoma (HepG2) and nasopharyngeal carcinoma (SUNE1).^[24]^ In the same study, Che and co‐workers identified that analogous mononuclear complexes, [(C^N^N)Pt(NHC)]^+^ displayed significantly higher antiproliferative activity, with nanomolar to sub‐micromolar potency (IC_50_=0.057–1.3 μm), compared to the dinuclear [((C^N^N)Pt)_2_(*μ*‐NHC)]^+^ platinum(II) complexes, (IC_50_=3.9–9.4 μm) against three tested cancer cell lines (HeLa, HepG2 and SUNE1).^[24]^ Lowe and co‐workers investigated the influence of linker rigidity on the cytotoxicity of a series of binuclear [((N^N^N)Pt)_2_(bipy)] (where N^N^N=terpy and bipy is a range of pyridine substituted derivatives connected by different linker groups) against several human ovarian cell lines including those resistant to cisplatin and doxorubicin; CH1, CH1cis, CH1dox, A2780 and A2780cisR. Generally, they found that the presence of short rigid linkers, for example, 4,4‘‐bipyridine (4,4‘‐bipy), between the metal centres generated complexes with potent cytotoxicity against the tested cancer cell lines including nanomolar IC_50_ values against the doxorubicin resistant ovarian cell line CH1dox.^[12a]^ It is evident from the studies by Che and Lowe that the cytotoxicity of dinuclear cyclometallated platinum(II) complexes is highly dependent on several factors, including the backbone of the structure and the nature of the linker ligand. However, despite the increasing importance of cyclometallated platinum(II)‐based complexes as potential anticancer therapeutics, reports on systematic studies to elucidate SARs for this class of compounds are limited.

**Figure 1:**
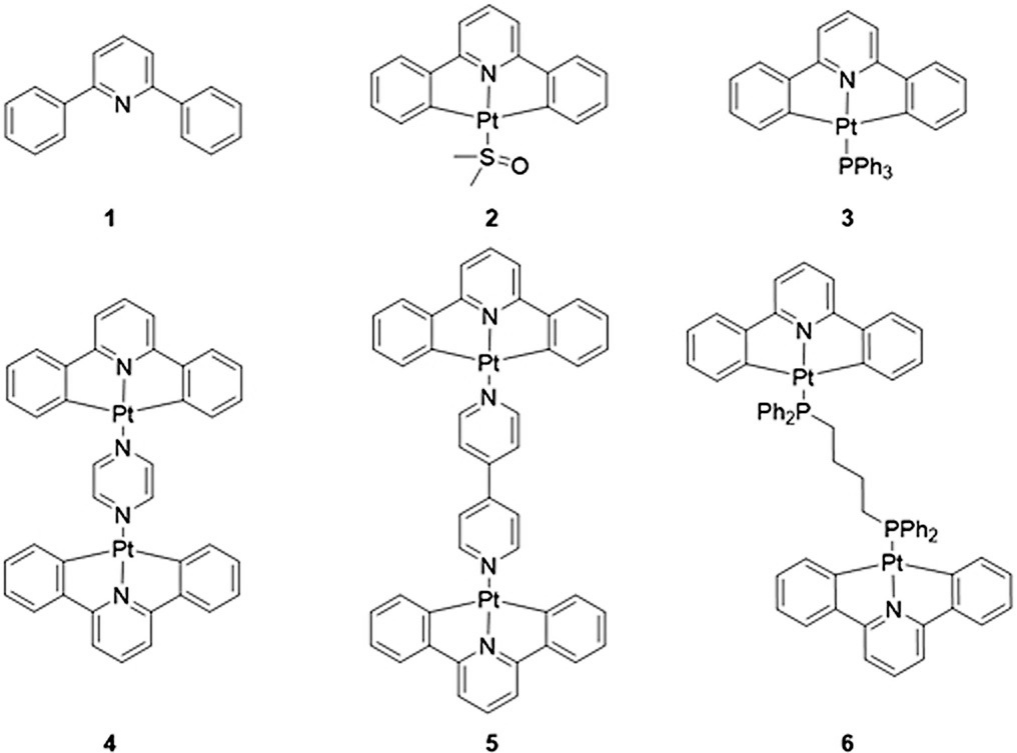
Library of compounds employed in this study. Ligand **1**, mononuclear complexes **2** and **3** and dinuclear complexes **4**–**6**.

In this study, we investigate the SARs for a series of mono‐ and di‐nuclear cyclometallated C^N^C platinum(II) compounds (Figure 1), [(C^N^C)Pt^II^(**L**)] (**L**=dimethylsulfoxide (DMSO) **2** and triphenylphosphine (PPh_3_) **3**) and [((C^N^C)Pt^II^)_2_(**L‘**)] (**L‘**=*N*‐heterocycles (pyrazine (pyr) **4**, 4,4‘‐bipyridine (4,4‘‐bipy) **5** or diphosphine (1,4‐bis(diphenylphosphino)butane (dppb) **6**) respectively, (where HC^N^CH=diphenylpyridine), against a range of cancer cell lines. This series of cyclometallated platinum(II) complexes were designed to consider the influence of three important structural features; 1) changes to the nature of the donor atom at the fourth coordination site of the metal centre (i.e., **L** and **L‘**) (from sulfoxide, phosphines and *N*‐heterocycles), 2) size of ligand spacer between the two metal centres for two dinuclear complexes (from pyrazine (pyr) to 4,4‘‐bipy) and 3) nuclearity of the complex (from mono‐ and di‐nuclear). We also report chemosensitivity studies against a normal cell type, showing that the most promising candidate, [((C^N^C)Pt^II^)_2_(dppb)], is non‐toxic towards normal cells, unlike cisplatin, which demonstrates high cytotoxicity.

## Results and Discussion

### Design and synthesis of library of compounds

The library of compounds consists of a ligand, 2,6‐diphenylpyridine **1** (HC^N^CH), two mononuclear complexes, [(C^N^C)Pt(DMSO)] **2** (where DMSO=dimethylsulfoxide) and [(C^N^C)Pt(PPh_3_)] **3** (where PPh_3_=triphenylphosphine), and three dinuclear platinum(II) complexes [((C^N^C)Pt)_2_(pyr)] **4**, [((C^N^C)Pt)_2_(4,4‘‐bipy)] **5** and [((C^N^C)Pt)_2_(dppb)] **6** (Figure 1). Compounds **1**–**6** were prepared using known or modified literature procedures.[^31^, ^32^, ^33^, ^34^] Compounds **1**–**5** were characterized by ^1^H NMR spectroscopy, mass spectrometry, FTIR spectroscopy and elemental analysis. Compound **1** was additionally characterized by ^13^C{^1^H} NMR spectroscopy.[^31^, ^32^, ^33^, ^34^] Compounds **2** and **3** were further characterized by single crystal X‐ray diffraction and both complexes were confirmed to crystallize in the same crystal space group as the published literature structures.[^31^, ^34^] Additionally, **6** was characterized by ^1^H, ^13^C{^1^H} and ^31^P{^1^H} NMR spectroscopy, mass spectrometry, melting point analysis, FTIR spectroscopy and single crystal X‐ray diffraction. A singlet resonance is observed in the ^31^P{^1^H} NMR spectrum of **6** at *δ* 20.3 ppm (J_PPt_=4960 Hz) and is due to the two phosphines coupling with the platinum centres in this symmetric molecule.

**Figure 2:**
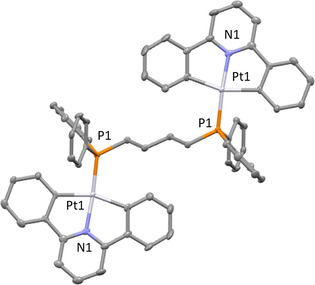
Molecular structure of complex **6**. Hydrogen atoms have been omitted for clarity and displacement ellipsoids are at the 50 % probability level. Pt−P: 2.2190(17)–2.2216(18) Å; Pt−N: 2.022(5)–2.0225(5) Å; and Pt−C: 2.076(6)–2.091(6) Å and bond angles (ranging from 79.5(2)–105.7(2)°). C atoms are shown in grey, N in light blue, Pt in white and P in orange.

Bright yellow crystals of **6** suitable for X‐ray diffraction were obtained through the slow evaporation of a concentrated chloroform solution at room temperature. The complex crystallizes in a monoclinic crystal system and solution refinement was performed in the space group *P*2_1_/*c* (Table S3 in Supporting Information). The molecular structure of **6** is shown in Figure 2, with displacement ellipsoids placed at the 50 % probability level and hydrogen atoms omitted for clarity. All of the bond lengths and angles are representative of a *pseudo* square‐planar geometry expected of d^8^ Pt^II^ complex (Tables S4 and S5).^[31]^ In the crystal packing of **6**, there are intermolecular, edge‐to‐face, π–π stacking interactions present between the phenyl rings of dppb and aromatics of the C^N^C pincer ligand (Figure S1).^[35]^

### Chemoselectivity studies

**Figure 3:**
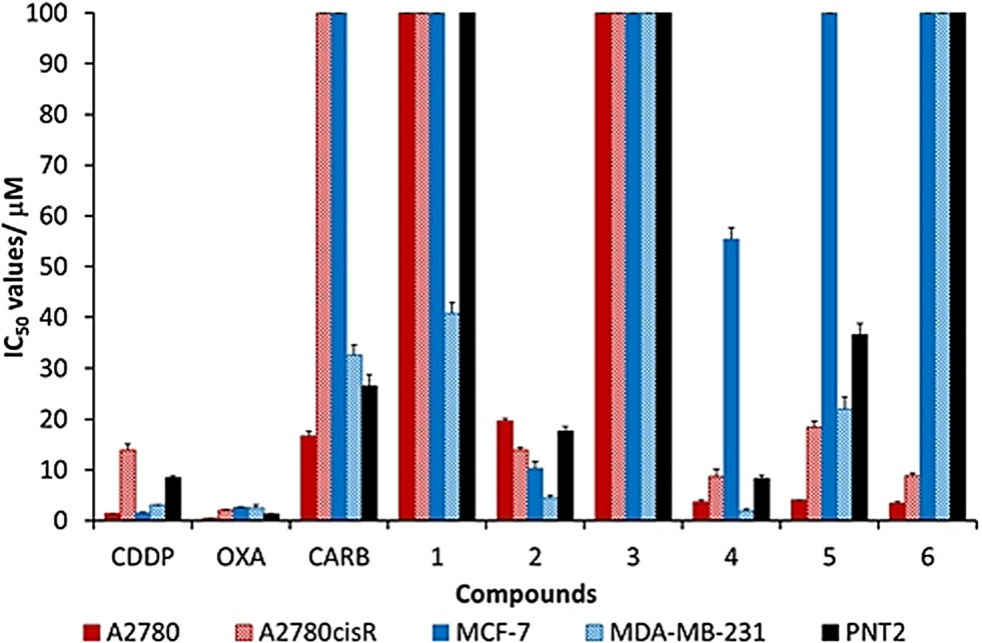
Cytotoxicity values (IC_50_/μm±SD) for cisplatin (**CDDP**), oxaliplatin (**OXA**), carboplatin (**CARB**), ligand **1** and complexes **2**–**6** against human ovarian cancer (A2780, A2780cisR), human breast cancer (MCF‐7, MDA‐MB‐231) and normal prostate cells (PNT2).

Ligand **1**, complexes **2**–**6**, cisplatin (**CDDP**), oxaliplatin (**OXA**) and carboplatin (**CARB**) were all screened for their cytotoxicity against human cell lines: cisplatin‐sensitive ovarian carcinoma (A2780), cisplatin‐resistant ovarian carcinoma (A2780cisR) and breast adenocarcinomas (MCF‐7 and MDA‐MB‐231). The IC_50_ values were obtained via the MTT assay after a 96 h incubation period of each compound with the cells at 37 °C and 5 % CO_2_ (Table 1 and Figure 3). Ligand **1** was found to be moderate to non‐toxic against all cell lines, with IC_50_ values of 41±2 μm to >100 μm. The mononuclear platinum(II) complexes, **2** and **3**, have significant differences in their cytotoxicity. The potency of complex **2** increases by up to 10‐fold against MCF‐7 in comparison to ligand **1** (*p*<0.05), and exhibits similar potency (IC_50_=4.4±0.5 μm) to **CDDP** (IC_50_=3.07±0.02 μm) against MDA‐MB‐231 (*p=*0.01). Importantly, **2** is the only compound in this library which has noticeable toxicity towards MCF‐7 (IC_50_=10±1 μm), however, it remains >6‐fold less cytotoxic than **CDDP** (*p*<0.05). A recent report by Klein and co‐workers determined that **2** has a IC_50_ value against MDA‐MB‐231 of 12.12±1.84 μm, which is within experimental error.^[21]^ Furthermore, **2** is more active towards the cisplatin‐resistant ovarian cell line A2780cisR than the parental cisplatin‐sensitive A2780 cells. On replacing the monodentate DMSO ligand in **2** for PPh_3_ in **3**, the activity significantly decreases, and **3** is non‐toxic against all cell lines tested (IC_50_>100 μm) (Table 1 and Figure 3).

**Table 1:** Cytotoxicity values (IC_50_/μm±SD) for cisplatin (**CDDP**), oxaliplatin (**OXA**), carboplatin (**CARB**), ligand **1** and complexes **2**–**6** after a 96 h incubation period with human ovarian carcinomas (A2780, A2780cisR), human breast adenocarcinomas (MCF‐7, MDA‐MB‐231) and normal prostate cells (PNT2).^[a]^ Selective Index (SI) values when compared to PNT2 are shown in parenthesis.

Compounds	IC_50_ values (μm)±SD				
	A2780	A2780cisR	MCF‐7	MDA‐MB‐231	PNT2
**CDDP**	1.3±0.1 (6.4)	14±1 (0.6)	1.5±0.2 (5.6)	3.07±0.02 (2.8)	8.5±0.4
**OXA**	0.505±0.002 (2.6)	2.09±0.03 (0.6)	2.6±0.2 (0.5)	2.5±0. 6 (0.5)	1.3±0.2
**CARB**	17±1 (1.6)	>100 (0.3)	>100 (0.3*)	33±2 (0.8)	27±2
**1**	>100 (n.d.)	>100 (n.d.)	>100 (n.d.)	41±2 (2.5*)	>100
**2**	19.7±0.5 (0.9)	13.9±0.5 (1.3)	10±1 (1.7)	4.4±0.5 (4.0)	18±1
**3**	>100 (n.d.)	>100 (n.d.)	>100 (n.d.)	>100 (n.d.)	>100
**4**	3.6±0.4 (2.3)	9±1 (1.0)	55±2 (0.2)	1.9±0.5 (4.4)	8.3±0.6
**5**	3.996±0.002 (9.2)	18±1 (2.0)	>100 (0.4*)	22±2 (1.7)	37±2
**6**	3.4±0.4 (29.5*)	8.9±0.5 (11.2*)	>100 (n.d.)	>100 (n.d.)	>100

[a] All values are averages from duplicate technical repeats and triplicate experimental repeats. * indicates the minimum SI value as at least one IC_50_ value is >100 μm. n.d. (not determined) indicates the values where both IC_50_ values are >100 μm.

Complex **4** was designed to study the cytotoxicity effects of a dinuclear system with the short pyrazine linker. The cytotoxicity of **4** increases by up to 27‐fold and 5‐fold against A2780 (*p*<0.05), in comparison to mononuclear complexes **2** and **3**, respectively. Upon extending the linker to 4,4‘‐bipy (**5**), the compound remains equitoxic to that of **4** against A2780, however, the toxicity decreases against all other cell lines, with up to a 2‐fold decrease against A2780cisR and an 11‐fold decrease against MDA‐MB‐231 (*p*<0.05). Harper et al. showed that the related [((N^N^N)Pt)_2_(4,4‘‐bipy)] (N^N^N=terpy) analogue of **5** has been reported to have GI_50_ values of 2.7±1.3 μm against A2780 and 5.6±0.4 μm against A2780cisR.^[29]^ Whilst Lowe and co‐workers reported that the same complex had IC_50_ values of >25 μm against A2780 and 9.6 μm against A2780cisR.^[12a]^ The differing IC_50_ values of **5** and the [((N^N^N)Pt)_2_(4,4‘‐bipy)] (N^N^N=terpy) analogue, highlights that the nature of the pincer ligand is also an important factor on determining the cytotoxicity of these cyclometallated platinum(II) complexes. On modification of the linker to a flexible butyl substituent, in the dinuclear diphosphine complex **6**, the cytotoxicity against MDA‐MB‐231 decreases even further, with >4‐fold decrease when compared to **5** and >52‐fold decrease when compared to **4**. Moreover, increasing the nuclearity of the platinum(II)‐phosphine compounds from mononuclearity in **3** to dinuclearity in **6** results in significantly increased cytotoxicity values against the ovarian carcinoma cell lines, with up to >29‐fold and >11‐fold increases observed against A2780 and A2780cisR, respectively. As the IC_50_ values of **3** against both ovarian cancer cell lines, A2780 and A2780cisR, is greater than the tested threshold concentration of 100 μm, this observed increase in cytotoxicity between these two platinum(II)‐phosphine ligands could be greater than reported here. As for **3**, complex **6** is non‐toxic against MCF‐7 and MDA‐MB‐231 (IC_50_>100 μm).

On analysis of these results, no definitive SARs can be determined, however, some important structural features can be highlighted; i) the nature of the ligand plays a significant role in determining the cytotoxicity of the mononuclear complexes, with the presence of the sulfoxide ligand in **2** increasing the potency in all test cancer cells lines by up to 25‐fold compared to the analogous phosphine‐platinum(II) complex **3** (e.g., **2** versus **3** against MDA‐MB‐231), ii) the addition of a second platinum centre and linker unit increases the potency of the compounds by up to 29‐fold (e.g. **3** versus **6** against A2780), iii) the shorter pyrazine linker in complex **4** is the optimal linker of the *N*‐heterocyclic dinuclear species exhibiting up to sub‐micromolar potency against MDA‐MB‐231 (IC_50_=1.9±0.5 μm). A notable result is that of complex **6**, which is non‐toxic towards either of the two human breast cancer cell lines (MCF‐7 and MDA‐MD‐231, IC_50_ >100 μm), has significantly increased cytotoxicity against the human ovarian cancer cell lines, >29‐fold against A2780 (IC_50_=3.4±0.4 μm, *p*<0.05) and >11‐fold against A2780cisR (IC_50_=8.9±0.5 μm, *p*<0.05).

### Selectivity index (SI)

Due to the current issues of the clinical platinum compounds (**CDDP**, **OXA** and **CARB**), which exhibit high potency towards normal cell types, this library of compounds was screened against normal prostate cell line, PNT2 (Table 1). As expected, the results highlight that the clinical platinum drugs are high to moderately cytotoxic towards this cell line, with IC_50_ values, 1.3±0.2 μm (**OXA**), 8.5±0.4 μm (**CDDP**) and 27±2 μm (**CARB**). Ligand **1** and complex **3** are non‐toxic towards PNT2 (IC_50_ >100 μm). Complexes **2**, **4** and **5** all remain moderately cytotoxic against the normal cell line, and even though complex **4** is >4‐fold more cytotoxic against MDA‐MB‐231 than PNT2, it remains relatively cytotoxic against normal cells (IC_50_=8.3±0.6 μm). The most promising result is observed for **6**, which remains non‐toxic against PNT2 (IC_50_>100 μm) yet is cytotoxic towards human ovarian carcinomas.

**Figure 4:**
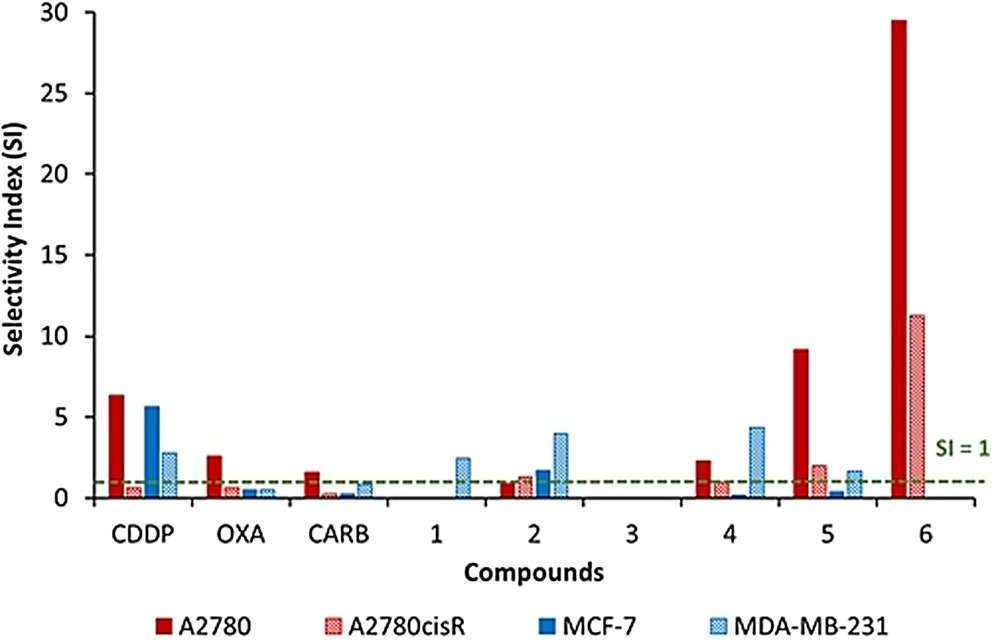
Selectivity Index (SI) for **CDDP**, **OXA**, **CARB**, ligand **1** and complexes **2**–**6** when the IC_50_ values of the cancerous lines are compared to those of the normal cell line PNT2. SI <1 indicates selectivity the normal cell line PNT2, SI=1 indicates equitoxicity and SI >1 indicates selectivity for the cancerous cell line.

The selectivity index (SI) values were calculated for all compounds, using the IC_50_ values obtained against PNT2 and dividing by the IC_50_ value against the cancer cell line (Table 1 and Figure 4). Whereby a SI value >1 indicates increases selectivity for the cancerous cell line over the normal cell line. Generally, compounds **1**–**4** do not have increased selectivity for A2780, A2780cisR and MCF‐7, however, there are some notable increases in selectivity for the hormone independent breast adenocarcinoma cell line, MDA‐MB‐231, where complexes **2** and **4** have SI of 4.0 and 4.4, respectively. Complex **5** displays a SI of 9.2 and 2.0 against the ovarian carcinoma cell lines, A2780 and A2780cisR respectively, which outperforms **CARB** by 5.8‐fold (A2780) and 6.7‐fold (A2780cisR) (Table 1). Unlike **4**, complex **5** does not have increased selectivity towards MDA‐MB‐231. The most promising result is observed for complex **6** against the ovarian cell lines, with SI values of >29.5 and >11.2 for A2780 and A2780cisR, respectively. These results are also minimum SI values, as the IC_50_ value against PNT2 is greater than the tested threshold concentration of 100 μm, and so the SI could be greater than reported here. Cisplatin has a high SI against A2780 (SI=6.4), however, **6** exhibits a SI value of 29.5, which is 4‐fold higher than that of **CDDP** (*p*<0.05). Importantly, the SI values observed for complex **6** against A2780cisR show a higher degree of selectivity than the clinical platinum compounds, with SI values >18‐fold (**CDDP** and **OXA**) and >42‐fold (**CARB**).

### Resistance and sensitivity factors

As many cancers become resistance to drugs, including the clinical platinum drugs, there is an urgent need to address the issues of drug resistance, by designing new and effective drugs. To address the potential of compounds **1**–**6** to target the ovarian cisplatin‐resistance cell line A2780cisR, the IC_50_ values were compared with those from the ovarian cisplatin‐sensitive cell line A2780. Resistance factors (RF)were obtained by dividing the IC_50_ values of A2780 by A2780cisR, where values >1 indicate a preference for the cisplatin resistant cell line A2780cisR. The RF values could not be calculated for ligand **1** and complex **3**, as IC_50_ values are >100 μm against both ovarian cell lines. As with the clinical platinum drugs, complexes **4**–**6** are all more cytotoxic towards A2780. However, complex **2** has a slightly higher selectivity for A2780cisR, with a RF value of 1.4 (Figure S2).

**Figure 5:**
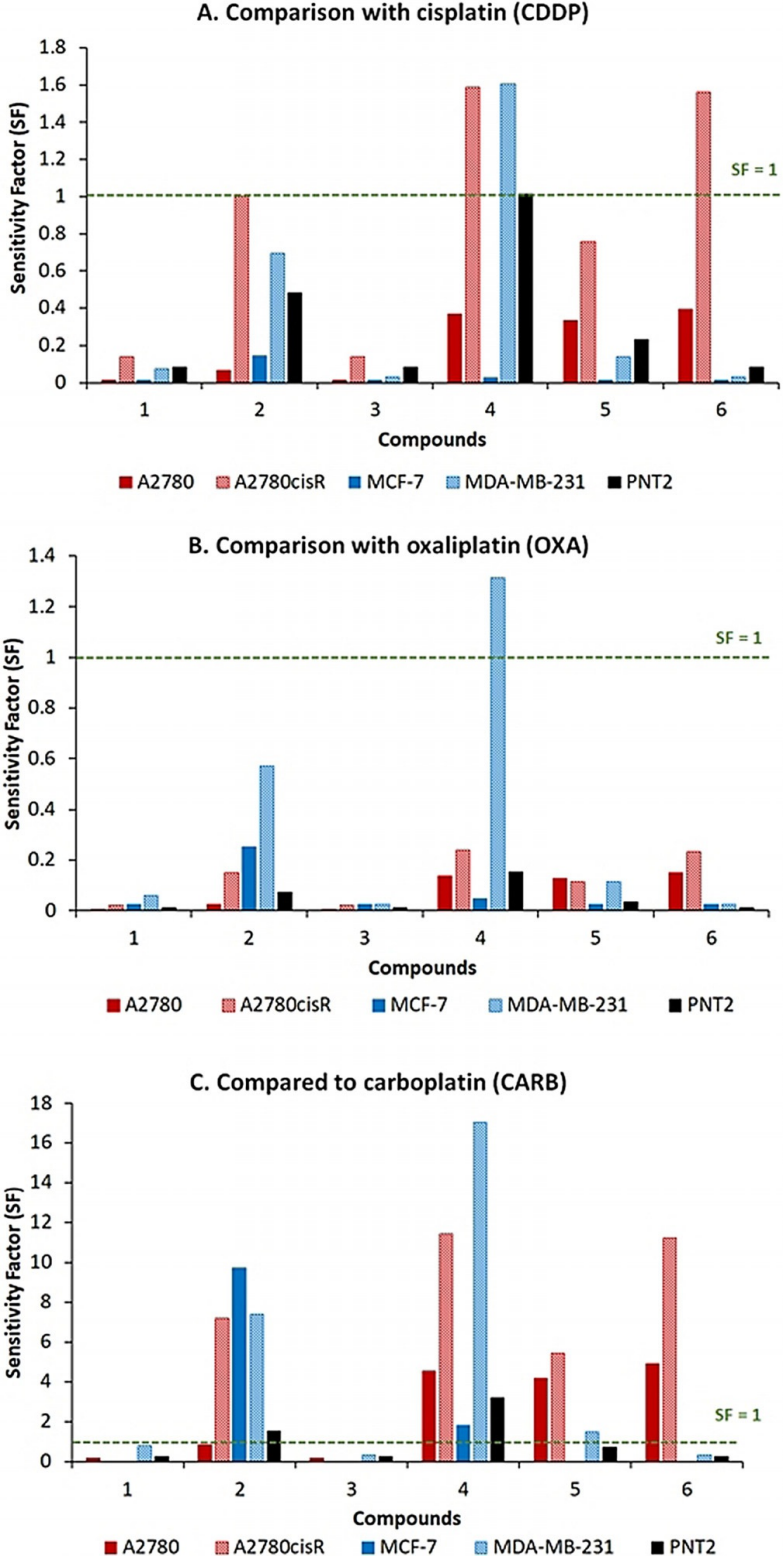
Sensitivity factor (SF) for ligand **1** and complexes **2**–**6** when the IC_50_ values are compared with A) cisplatin (**CDDP**), B) oxaliplatin (**OXA**) and C) carboplatin (**CARB**).

To address the activity of compounds **1**–**6** in comparison with the clinical drugs, the sensitivity factors (SF) were calculated by dividing the IC_50_ values of the clinical drugs by the IC_50_ values of compounds **1**–**6**. An SF >1 indicates a selectivity for our library of compounds over the clinical drugs (Figure 5). When comparing the performance of our library of compounds with **CDDP** (Figure 5 A), complexes **4** and **6** are 1.6x more cytotoxic against MDA‐MB‐231 (**4**) and A2780cisR (**4** and **6**) whilst the rest of the library displays SF<1 against the other tested cell lines. When the biological performance of **1**–**6** is compared to **OXA** (Figure 5 B), only complex **4** is more cytotoxic, and is 1.3x more active against MDA‐MB‐231. The most promising results are observed when comparing the cytotoxicity values of the library with **CARB**, wherein complexes **2**, **4**–**6** are markedly more cytotoxic than this clinical drug against a range of the tested cell lines (Figure 5 C). In particular, complexes **2** and **4** have high selectivity for MDA‐MB‐231, with IC_50_ values 7.4x and 17.0x higher than **CARB**, respectively. Triple negative breast cancers (TNBC) are the most complex and aggressive types of breast cancer,^[36]^ and are associated with high metastasis, patient relapse, poor prognosis and low survival rates. Therefore, designing and identifying drugs which are effective against these cancers is essential and urgent. Since several of our compounds have promising high activities against MDA‐MB‐231, this warrants further investigation into their use against TNBC. Complexes **2**, **4**–**6** all have increases activity towards one or more of the ovarian carcinomas, when compared to **CARB**, with significantly higher activity against A2780 displaying SF ranging from 4.3 (**5**) to 5 (**6**) and, notably, against the cisplatin‐resistance ovarian cell line A2780cisR, with SF ranging from 5.4 (**5**) to 11.4 (**4**). Additionally, complex **2** has significantly higher activity when compared to **CARB** against the breast adenocarcinoma MCF‐7, displaying an IC_50_ value at least 10x higher than this clinical drug.^[37]^

Together, these results highlight the significant cytotoxic potential for this library of compounds, especially for the dinuclear complexes **4** and **6**. Although complex **4** is moderately cytotoxic towards the normal cell line, its increased selectivity towards the TNBC line MDA‐MB‐231 is promising and should be investigated in further studies. Complex **6** is non‐toxic towards the normal cell line and has the highest cancer cell selectivity for this library, warranting further modifications and in‐depth *in vitro* studies. On analysis of these results, it is possible that these complexes have mechanism of actions which differs from that of the clinical platinum drugs. As it has previously been shown that cyclometallated N^N^N platinum(II) cationic complexes form strong intercalations with DNA, with binding constants from 0.8×10^5^ m ^−1^ to 2.0×10^7^ m ^−1^,[^38^, ^39^] it is possible that the compounds reported herein have the potential to exert their potency through a similar manner. In order to further develop this library of compounds, it is now necessary to obtained sufficient SARs and a more in‐depth *in vitro* screening, to underpin the cellular uptake and possible mechanisms of action of such diplatinum species.

## Conclusions

In conclusion, we have synthesized a library of mono‐ and di‐nuclear, cyclometallated platinum(II) complexes, of the general formula, [(C^N^C)Pt^II^(**L**)] and [((C^N^C)Pt^II^)_2_(**L‘**)] respectively, where H C^N^CH is 2,6‐diphenylpyridine, **L** is a monodentate ligand (DMSO **2** or PPh_3_ **3**) and **L‘** is a bidentate ligand (pyr **4**, 4,4‘‐bipy **5** or dppb **6**). All compounds were screened against human ovarian carcinomas (A2780 and A2780cisR), human breast adenocarcinomas (MCF‐7 and MDA‐MB‐231) and normal prostate cell line (PNT2). There are no definite conclusions which can be drawn on the SARs however, some general trends can be observed.

Firstly, comparing the mononuclear complexes, platinum(II)‐sulfoxide **2** and platinum(II)‐phosphine **3**, the nature of the monodentate ligand at the fourth coordination site of the metal centre strongly influences the antiproliferative activity of the complex. The former displaying significantly increased potency against all tested cancer cell lines (IC_50_=4.4–19.7 μm) compared to **3** which is non‐toxic (IC_50_>100 μm).

Secondly, shortening the length of the linker between the two metal centres in these cyclometallated platinum(II) complexes from pyr in **4** to 4,4‘‐bipy in **5**, has a significant effect on increasing the cytotoxicity of the complex, by up to 11‐fold, against three of the tested cancer cell lines (A2780cisR, MCF‐7 and MDA‐MB‐231).

Thirdly, there is a general increase in potency observed for the dinuclear complexes **4**–**6** when compared to the mononuclear complexes **2** and **3**. For the platinum(II)‐phosphine complexes **3** and **6**, the latter dinuclear complex showed significantly increased cytotoxicity against the ovarian carcinoma cell lines with up to >29‐fold and >11‐fold increase observed against A2780 and A2780cisR, respectively when compared to mononuclear complex **3**.

Several of the studied complexes are more cytotoxic than the clinical drug, carboplatin (**CARB**), with cytotoxicity values up to 7.4x (**2**) and 17.0x (**4**) against the TNBC line, MDA‐MB‐231. This is important in the development of breast cancer drugs, as this is the most complex and aggressive form of breast cancer. Generally, these cyclometallated complexes have increased activity towards ovarian carcinomas (compared to **CARB**) with significantly higher activity against the cisplatin‐resistance ovarian cell line A2780cisR, with sensitivity factors (SF) ranging from 5.4 (**5**) to 11.4 (**4**). Notably, complex **6** was non‐toxic towards the normal cell line (IC_50_>100 μm) and has selectivity index (SI) values >29 against A2780 and is up to 42‐fold more selective than current clinical platinum drugs.

We have identified the dinuclear platinum(II)‐phosphine complex **6** as the lead candidate of the studied library, as it remains non‐toxic towards the normal cell line, and our future work will now be aimed at underpinning the specific mechanisms of action of this complexes. We anticipate that the insights gained from this systematic study will continue to help inform the future design of novel cyclometallated diplatinum(II) complexes with high in vitro potential.

## Experimental Section

**General experimental details**: All NMR spectroscopy was carried out on a Bruker Advance 400 FT NMR spectrometer using the residual solvent as the internal standard. All the chemical shifts (δ) are quoted in ppm and coupling constants are given in Hz and are rounded to 0.1 Hz. Melting points were obtained on GallenKamp and are uncorrected. Single‐crystal X‐ray diffraction data on **2**, **3** and **6** was collected using a Bruker X8 diffractometer with an APEX II detector and monochromated Mo_Kα_ radiation (*λ*=0.7107 Å) at 173 K. The data was processed using Bruker SAINT, the structures determined with SHELXT^[40]^ and subsequently refined with SHELXL^[41]^ within the program olex2.^[42]^ Crystal structures were visualized using Mercury.^[43]^ Infrared spectroscopy was carried out on a PerkinElmer 100 FTIR instrument fitted with an ATR detector. Mass spectrometry was recorded on a Waters Micromass Quattro Ultima quadrupole mass spectrometer at the Bradford Analytical Centre. All the reactions were conducted under nitrogen atmosphere. Potassium carbonate, sodium carbonate, concentrated aqueous ammonia and acetic acid were purchased from Camlab. Dimethylsulfoxide, toluene, methanol, dichloromethane, petroleum ether, di‐ethyl ether and chloroform were purchased from Fisher. Tetrakis(triphenylphosphine)palladium(0), 2,6‐dibromopyridine, triethylamine, 4‐phenylboronic acid, pyrazine, 4,4‘‐bipyridine, triphenylphosphine, 1,4‐bis(diphenylphosphino)butane and potassium tetrachloroplatinate were purchased from Sigma Aldrich. Petrol refers to the fraction of light petroleum ether boiling between 40 and 60 °C. All chemicals were used as received unless otherwise stated. The following abbreviations are employed: 4,4‘‐bipy=4,4‘‐bipyridine, aq.=aqueous, Ar=aromatic, br=broad, calc.=calculated, CARB=carboplatin, CDDP=cisplatin, d=doublet, DMSO=dimethylsulfoxide, dppb=1,4‐bis(diphenylphosphino)butane, eq.=equivalent(s), Et=ethyl, h=hour(s), Hz=Hertz, IC_50_=half maximal inhibitory concentration, IR=infrared, m=multiplet, Me=methyl, m.p.=melting point, MTT=3‐(4,5‐dimethylthiazol‐2‐yl)‐2,5‐diphenyltetrazolium bromide, NMR=nuclear magnetic resonance, OXA=oxaplatin, pet.=petroleum, Ph=phenyl, pyr=pyrazine, s=singlet, SD=standard deviation, SI=selectivity index, terpy=terpyridine, TNBC=triple negative breast cancer and t=triplet. Compounds **1**–**5** were synthesized according to known literature procedures.[^31^, ^32^, ^33^]

**Cell culture**: *In vitro* chemosensitivity tests were performed against cisplatin‐sensitive human ovarian carcinoma (A2780), cisplatin‐resistant human ovarian carcinoma (A2780cis) and human breast adenocarcinomas (MCF‐7 and MDA‐MB‐231). Additionally, growth inhibitory effects were also tested against normal prostate cell line, PNT2. All cell lines were provided by the Institute of Cancer Therapeutics, University of Bradford and were routinely maintained as monolayer cultures in RPMI 1640 media supplemented with 10 % foetal calf serum, sodium pyruvate (1 mm) and l‐glutamine (2 mm). All assays were conducted in 96‐well round bottom plates, with control lanes for media and 100 % cell growth. Cell concentrations of 1×10^4^ cells mL^−1^ were used, and 100 μL (or 100 μL media in control lane 1) of cell suspension were incubated for 24 hours at 37 °C and 5 % CO_2_ prior to drug exposure. Ligand **1**, complexes **2**–**6**, cisplatin (**CDDP**), oxaliplatin (**OXA**) and carboplatin (**CARB**) were all freshly dissolved in DMSO to provide 100 mm stock solutions, which were further diluted with complete media to provide a range of final concentrations. After 24 hours incubation, 100 μL of the drug/media solutions were added to the plates in columns 3–12 (100 μL media in lanes 1 and 2 for controls), and then the plates incubated for 96 hours at 37 °C and 5 % CO_2_. Drug solutions were added to cells so that the final DMSO concentrations were less than 0.1 % (v/v) in all cases. After 96 hours, 3‐(4,5‐dimethylthiazol‐2‐yl)‐2,5‐diphenyltetrazolium bromide (MTT) (20 μL, 5 mg mL^−1^) was added to each well and incubated for 3 hours at 37 °C and 5 % CO_2_. All solutions were then removed via pipette and 150 μL DMSO added to each well in order to dissolve the purple formazan crystals. A Thermo Scientific Multiscan EX microplate photometer was used to measure the absorbance of each well at 540 nm. Percentage cell viabilities were determined on a logarithmic scale, and the half maximal inhibitory concentration (IC_50_) determined from a plot of % cell survival versus concentration (μm). Each of the experiments was performed as duplicate technical repeats and triplicate experimental repeats, with mean values stated as IC_50_ ± Standard Deviation (SD).

**Statistical analysis**: A two‐tailed ANOVA t‐test has been conducted using Graph Pad Prism 8, and used to compare all chemosensitivity data: probability values *p*<0.05 are considered significant.

### Synthetic details

**1**: A solution of 2,6‐dibromopyridine (2.0 g, 8.4 mmol) and Pd(PPh_3_)_4_ (0.39 g, 0.34 mmol) in toluene (28 mL) was treated with a solution of Na_2_CO_3_ (3.6 g, 34 mmol) in H_2_O (17 mL). A solution of 4‐phenylboronic acid (4.1 g, 34 mmol) in methanol (35 mL) was then added to this two‐phase system and the reaction mixture stirred at 85 °C for 16 h. Upon cooling to room temperature, concentrated aqueous NH_3_ (4 mL) and sat. Na_2_CO_3_ (40 mL) were added and the aq. phase was extracted with CH_2_Cl_2_ (3×30 mL). The organic washings were combined and washed with brine, dried over MgSO_4_ and concentrated down under reduced pressure. The resulting residue was subjected to flash column chromatography (2:98; Et_2_O:pet. ether) and crystals were grown through recrystallization from methanol and Et_2_O. (1.9 g, 77 %). ^1^H NMR (400 MHz, CDCl_3_, 298 K): *δ* 8.17–8.14 (4 H, m, Ar*H*), 7.83 (1 H, t, ^3^ *J*=8.0 Hz, Ar*H*), 7.70 (2 H, dd, ^3^ *J*=8.0 Hz, ^4^ *J*=0.4 Hz, Ar*H*), 7.53–7.48 (4 H, m, Ar*H*), 7.45–7.41 (2 H, m, Ar*H*), ^13^C{^1^H} NMR (100 MHz, CDCl_3_, 298 K): *δ* 156.8, 139.5, 137.5, 129.0, 128.7, 127.0, 118.6; IR (solid) *ν*_max_ 3058 (w), 3027 (w), 1573 (m), 1562 (m) cm^−1^; LR‐ESIMS (+ve) *m*/*z* (%): 232 [*M*+H]^+^ (100 %); HR‐ESIMS (+ve): calcd 232.1121 found 232.1115 for C_17_H_14_N. Elemental Analysis: Anal. Found: C: 88.2, H: 5.7, N: 6.1 %. Anal. Calculated: C: 88.3, H: 5.7, N: 6.1 %.

**2**: To a solution of H_2_ **L** (0.30 g, 1.3 mmol) in acetic acid (55 mL) was added a solution of K_2_PtCl_4_ (0.54 g, 1.3 mmol) in H_2_O (3 mL) in a dropwise manner to generate a light rose coloured solution. The mixture was refluxed for 18 h, after which time, the red colour of the Pt salt had disappeared. The yellow precipitate that had formed was filtered off and washed with H_2_O (4 mL), acetone (4 mL), Et_2_O (4 mL) and pet. ether (2 mL). The yellow solid was then dissolved in DMSO (3 mL) and K_2_CO_3_ (0.75 g, 5.4 mmol) and H_2_O (2 mL) were added and the mixture heated to 90 °C for 1 h. After this time, the reaction mixture was allowed to cool to room temperature and upon the addition of H_2_O (10 mL), the product precipitated out as a bright yellow solid. This crude product was subjected to flash column chromatography (CH_2_Cl_2_). Single bright yellow crystals suitable for X‐ray diffraction were grown through the slow evaporation of chloroform from a saturated solution of **2**. (0.27 g, 41 %). ^1^H NMR (400 MHz, CDCl_3_, 298 K): *δ* 7.81 (2 H, dd, ^3^ *J*=7.2 Hz, ^4^ *J*=0.8 Hz, J(^195^Pt)=29.6 Hz, Ar*H*), 7.63 (1 H, t, ^3^ *J*=8.0 Hz, Ar*H*), 7.49 (2 H, dd, ^3^ *J*=7.6 Hz, ^4^ *J*=1.2 Hz, Ar*H*), Ar*H*), 7.33–7.27 (4 H, m, Ar*H*), 7.13 (2 H, dt, ^3^ *J*=7.2 Hz, ^4^ *J*=1.2 Hz, Ar*H*), 3.69 (6 H, s, J(^195^Pt)=27.2 Hz, 2 × CH_3_); IR (solid) *ν*_max_ 3034 (w), 2920 (w), 1598 (m), 1577 (m), 1562 (m) cm^−1^; LR‐ESIMS (+ve) *m*/*z* (%): 503 [*M*+H]^+^(100 %); HR‐ESIMS (+ve): calcd 503.0751 found 503.0745 for C_19_H_18_NO_2_NaPtS. Elemental Analysis: Anal. Found: C: 45.6, H: 3.3, N: 2.8 %. Anal. Calculated: C: 45.4, H: 3.4, N: 2.8 %.

**3**: To a solution of **2** (0.050 g, 0.10 mmol) in CH_2_Cl_2_ (2 mL) was added PPh_3_ (0.026 g, 0.10 mmol) and the resultant pale‐yellow solution was stirred at ambient temperature for 1 min. After this time, the excess solution was removed under reduced pressure and the resulting yellow residue was subjected to flash column chromatography (1:1:0.01; CH_2_Cl_2_:pet. ether:NEt_3_) to yield a bright yellow solid. Single crystals suitable for X‐ray crystallography were grown through the slow diffusion of di‐ethyl ether into a saturated solution of **3** in chloroform. (0.025 g, 37 %). ^1^H NMR (400 MHz, [D_6_]DMSO, 298 K): *δ* 7.94 (1 H, t, ^3^ *J*=7.6 Hz, Ar*H*), 7.85–7.80 (6 H, m, Ar*H*), 7.73 (2 H, dd, ^3^ *J*=8.4 Hz, ^4^ *J*=1.6 Hz, Ar*H*), 7.62 (2 H, dd, ^3^ *J*=8.0 Hz, ^4^ *J*=1.2 Hz, Ar*H*), 7.60–7.51 (9 H, m, Ar*H*), 6.93 (2 H, dt, ^3^ *J*=7.2 Hz, ^4^ *J*=1.2 Hz, Ar*H*), 6.62 (2 H, dt, ^3^ *J*=7.6 Hz, ^4^ *J*=1.6 Hz, Ar*H*), 6.18 (2 H, d, ^3^ *J*=7.6 Hz, J(^195^Pt)=32.4 Hz, Ar*H*); IR (solid) *ν*_max_ 3040 (w), 1597 (m), 1563 (m), 1508 (w) cm^−1^; LR‐ESIMS (+ve) *m*/*z* (%): 687 [*M*+H]^+^(100 %); Elemental Analysis: Anal. Found: C: 61.1, H: 3.7, N: 2.1 %. Anal. Calculated: C: 61.2, H: 3.8, N: 2.0 %.

**4**: To a solution of **2** (0.050 g, 0.10 mmol) in CH_2_Cl_2_ (2 mL) was added pyrazine (0.039 g, 0.050 mmol) and the resultant pale‐yellow solution was stirred at ambient temperature overnight. After this time, the red precipitate that had formed was filtered off, washed with CH_2_Cl_2_ (2 mL) to give the desired product as a bright red solid. (0.002 g, 4 %). ^1^H NMR (400 MHz, [D_6_]DMSO, 298 K): *δ* 8.65 (4 H, s, Ar*H*), 7.84 (2 H, t, ^3^ *J*=8.4 Hz, Ar*H*), 7.76 (4 H, dd, ^3^ *J*=7.2 Hz, ^4^ *J*=0.8 Hz, Ar*H*), 7.65–7.63 (8 H, m, Ar*H*), 7.19 (4 H, dt, *J*=7.6 Hz, ^4^ *J*=1.2 Hz, Ar*H*), 7.08 (4 H, d, ^3^ *J*=7.6 Hz, Ar*H*); IR (solid) *ν*_max_ 3043 (w), 1697 (w), 1598 (m), 1575 (m), 1560 (m) cm^−1^; LR‐ESIMS (+ve): 929 [*M*+H]^+^ (100 %); Elemental Analysis: Anal. Found: C: 49.0, H: 2.8, N: 6.0 %. Anal. Calculated: C: 49.1, H: 2.8, N: 6.0 %.

**5**: To a solution of **2** (0.050 g, 0.10 mmol) in CH_2_Cl_2_ (2 mL) was added 4,4‘‐bipyridine (0.0080 g, 0.050 mmol) and the resultant pale‐yellow solution was stirred at ambient temperature overnight. After this time, the red precipitate that had formed was filtered off, washed with CH_2_Cl_2_ (2 mL) to give the desired product as a bright orange solid. (0.03 g, 58 %). ^1^H NMR (400 MHz, [D_6_]DMSO, 298 K): *δ* 8.73 (4 H, dd, ^3^ *J*=6.0 Hz, ^4^ *J*=0.4 Hz, Ar*H*), 7.84–7.82 (6 H, m, Ar*H*), 7.77 (4 H, dd, ^3^ *J*=7.6 Hz, ^4^ *J*=1.2 Hz, Ar*H*), 7.66–7.63 (8 H, m, Ar*H*), 7.20 (4 H, dt, ^3^ *J*=7.6 Hz, ^4^ *J*=1.6 Hz, Ar*H*), 7.08 (4 H, dt, ^3^ *J*=7.6 Hz, ^4^ *J*=1.2 Hz, Ar*H*); IR (solid) *ν*_max_ 3041 (w), 1597 (m), 1575 (m), 1558 (m), 1541 (m) cm^−1^; LR‐ESIMS (+ve): 1005 [*M*+H]^+^; Elemental Analysis: Anal. Found: C: 51.9, H: 3.0, N: 5.4 %. Anal. Calculated: C: 52.6, H: 3.0, N: 5.6 %.

**6**: To a solution of **2** (0.050 g, 0.095 mmol) in CH_2_Cl_2_ (2 mL) was added dppb (0.021 g, 0.048 mmol) and the resultant pale‐yellow solution was stirred at ambient temperature for 1 h. After this time, the pale‐yellow precipitate that had formed was filtered off and yellow crystals suitable for X‐ray diffraction were grown through the slow diffusion of pet. ether into a saturated solution of chloroform (0.015 g, 12 %). m.p. 310–311 °C; ^1^H NMR (400 MHz, CD_2_Cl_2_, 298 K): *δ* 7.70–7.65 (10 H, m, Ar*H*), 7.44 (4 H, dd, ^3^ *J*=8 Hz, ^4^ *J*=1.2 Hz, Ar*H*), 7.38–7.28 (16 H, m, Ar*H*), 6.93 (4 H, dt, ^3^ *J*=8 Hz, ^4^ *J*=1.2 Hz, Ar*H*), 6.70 (4 H, dt, *J*=8 Hz, ^4^ *J*=1.2 Hz, Ar*H*), 6.55 (4 H, d, ^3^ *J*=8 Hz, ^195^ *J*=12 Hz, Ar*H*), 2.62–2.55 (4 H, s, C*H*_2_), 2.01–1.99 (4 H, s, C*H*_2_); ^13^C{^1^H} NMR (100 MHz, CD_2_Cl_2_, 298 K): *δ* 165.7 (J_C‐Pt_=104 Hz), 150.2, 139.6, 138.0 (J_C‐Pt_=116 Hz), 132.6, 132.5 (J_C‐Pt_=100 Hz), 129.5, 129.1, 127.5, 127.4, 123.2, 122.7, 114.2, 25.7, 25.3; ^31^P NMR (162 MHz, CD_2_Cl_2_, 298 K): *δ* 20.3 (J_P‐Pt_=4960 Hz); IR (solid) *ν*_max_ 3047 (w), 2936 (w), 2918 (w), 1597 (m), 1578 (m), 1565 (m), 1546 (m); LR‐ESIMS (+ve): 1275 [*M*+H]^+^; 1297 [M+Na]^+^; HRESI (+ve): calcd 1275.2823 found 1275.2803 for C_62_H_51_N_2_P_2_Pt_2_; calcd 1297.2642 found 1297.2637 for C_62_H_50_N_2_NaP_2_Pt_2_.

Deposition Number(s) 1998373, 1998377 and 1998378 contain(s) the supplementary crystallographic data for this paper. These data are provided free of charge by the joint Cambridge Crystallographic Data Centre and Fachinformationszentrum Karlsruhe Access Structures service www.ccdc.cam.ac.uk/structures.

## Conflict of interest

The authors declare no conflict of interest.

## Supporting information

As a service to our authors and readers, this journal provides supporting information supplied by the authors. Such materials are peer reviewed and may be re‐organized for online delivery, but are not copy‐edited or typeset. Technical support issues arising from supporting information (other than missing files) should be addressed to the authors.

Supplementary
