# Supplementary material for: Influence of Ligand and Nuclearity on the Cytotoxicity of Cyclometallated C^N^C Platinum(II) Complexes
Source: Chemistry. 2020 Oct 15;26(65):14938–46. doi: 10.1002/chem.202002517 (PMC7756510; doi:10.1002/chem.202002517)
Supplement: Supplementary file 1 — Supplementary [file CHEM-26-14938-s001.pdf]

Chemistry–A European Journal

Supporting Information

Influence of Ligand and Nuclearity on the Cytotoxicity of Cyclometallated C^N^C Platinum(II) Complexes

Angélique Kergreis,[a] Rianne M. Lord,*[a, b] and Sarah J. Pike*[a, c]

Supporting Information

[a]School of Chemistry and Biosciences, Faculty of Life Sciences, University of Bradford, Bradford, West Yorkshire, BD7 1DP, U.K.

[b]School of Chemistry, University of East Anglia, Norwich Research Park, Norwich, NR4 7TJ, U.K.

[c]School of Chemistry, University of Birmingham, Edgbaston, Birmingham, B15 2TT, U.K.

Email: R.Lord@uea.ac.uk, s.j.pike@bham.ac.uk

Contents *S2*

General Experimental Details *S3*

Cell Culture *S3*

Crystal data and structure refinement for [(C^N^C)Pt(DMSO)] *S5*

Crystal Data and Structural Refinement for [(C^N^C)Pt(PPh_3)] *S6*

Crystal data and structure refinement for [((C^N^C)Pt)$_2$(dppb)] *S7*

Solid-State Analysis of [((C^N^C)Pt)$_2$(dppb)] *S11*

Chemosensitivity Studies *S12*

Appendix: NMR spectra *S13*

References *S20*

General Experimental Details

All NMR spectroscopy was carried out on a Bruker Avance 400 FT NMR spectrometer using the residual solvent as the internal standard. Single crystal X-ray diffraction data on [(C^N^C)Pt(DMSO)],[1] [(C^N^C)Pt(PPh_3)][2] and [((C^N^C)Pt)$_2$(dppb)] was collected using a Bruker X8 diffractometer with an APEX II detector and monochromated Mo Kα radiation (λ = 0.7107 Å) at 173 K. The data was processed using Bruker SAINT, the structures determined with SHELXT[3] and subsequently refined with SHELXL[4] within the program olex2.[5] Crystal structures were visualised using Mercury.[6] The following abbreviations are employed: 4,4`-bipy = 4,4`-bipyridine, CARB = carboplatin, CDDP = cisplatin, DMSO = dimethylsulfoxide, dppb = 1,4-bis(diphenylphosphino)butane, IC_{50} = half maximal inhibitory concentration, MTT = 3-(4,5-dimethylthiazol-2-yl)-2,5-diphenyltetrazolium bromide, OXA = oxaplatin, Ph = phenyl, pyr = pyrazine, SD = standard deviation and SI = selectivity index.

Cell culture

In vitro chemosensitivity tests were performed against human ovarian carcinoma (A2780), cisplatin-resistant human ovarian carcinoma (A2780cis) and human breast adenocarcinomas (MCF-7 and MDA-MB-231). Additionally, growth inhibitory effects were also tested against normal prostate cell line, PNT2. All cell lines were provided by the Institute of Cancer Therapeutics, University of Bradford and were routinely maintained as monolayer cultures in RPMI 1640 media supplemented with 10% foetal calf serum, sodium pyruvate (1 mM) and L-glutamine (2 mM). All assays were conducted in 96-well round bottom plates, with control lanes for media and 100% cell growth. All of the cell culture undertaken at the University of Bradford is

ethically approved. Cell concentrations of 1 x 10^4 cells/mL were used, and 100 μL (or 100 μL media in control lane 1) of cell suspension were incubated for 24 hours at 37 °C and 5% CO_2 prior to drug exposure. Ligand **1**, complexes **2-6**, cisplatin, oxaliplatin and carboplatin were all dissolved in dimethylsulfoxide (DMSO) to provide 100 mM stock solutions, which were further diluted with complete media to provide a range of final concentrations. After 24 hours incubation, 100 μL of the drug/media solutions were added to the plates in columns 3-12 (100 μL media in lanes 1 and 2 for controls), and then the plates incubated for 96 hours at 37°C and 5% CO_2. Drug solutions were added to cells so that the final DMSO concentrations were less than 0.1% (v/v) in all cases. After 96 hours, 3-(4,5-dimethylthiazol-2-yl)-2,5-diphenyltetrazolium bromide (MTT) (20 μL, 5 mg/mL) was added to each well and incubated for 3 hours at 37 °C and 5% CO_2. All solutions were then removed via pipette and 150 μL DMSO added to each well in order to dissolve the purple formazan crystals. A Thermo Scientific Multiskan EX microplate photometer was used to measure the absorbance of each well at 540 nm. Percentage cell viabilities were determined on a logarithmic scale, and the half maximal inhibitory concentration (IC_{50}) determined from a plot of % cell survival versus concentration (μM). Each of the experiments was performed as duplicate technical repeats and triplicate experimental repeats, with mean values as the IC_{50} ± Standard Deviation (SD).

Crystal Data and Structural Refinement for [(C^N^C)Pt(DMSO)]

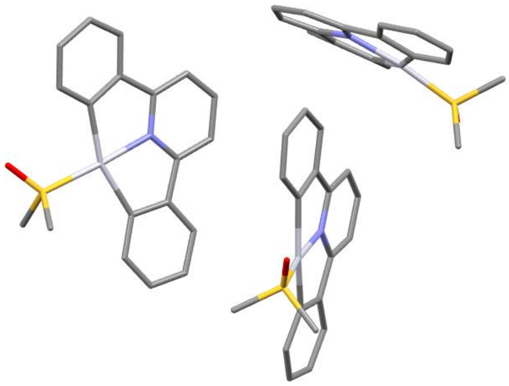

Table 1 Crystal data and structure refinement for [(C^N^C)Pt(DMSO)]

Identification code	[(C^N^C)Pt(DMSO)]
Empirical formula	$C_{19}H_{17}NOPtS$
Formula weight	502.49
Temperature/K	293(2)
Crystal system	monoclinic
Space group	$P2_1/c$
a/Å	17.797(4)
b/Å	9.916(2)
c/Å	27.593(6)
α/°	90
β/°	97.62(3)
γ/°	90
Volume/$Å^3$	4826.4(17)
Z	12
ρ_{calc}g/cm^3	2.075
μ/mm^{-1}	8.855
F(000)	2880.0
Crystal size/mm^3	0.34 × 0.23 × 0.17
Radiation	MoKα (λ = 0.71073)
2Θ range for data collection/°	4.85 to 55.036
Index ranges	$-23 \leq h \leq 23$, $-12 \leq k \leq 12$, $-35 \leq l \leq 35$
Reflections collected	68379
Independent reflections	11074 [R_{int} = 0.1347, R_{sigma} = 0.0763]
Data/restraints/parameters	11074/600/628
Goodness-of-fit on F^2	0.978
Final R indexes [I>=2σ (I)]	R_1 = 0.0443, wR_2 = 0.1044
Final R indexes [all data]	R_1 = 0.0594, wR_2 = 0.1094
Largest diff. peak/hole / e $Å^{-3}$	4.49/-2.96

Crystal Data and Structural Refinement for [(C^N^C)Pt(PPh_3)]

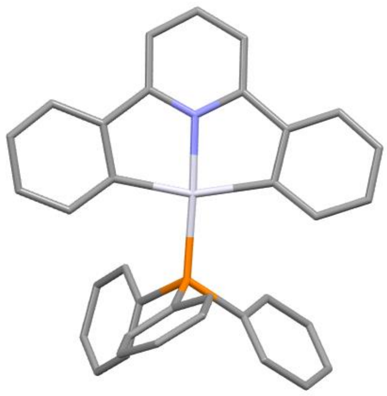

Table 2 Crystal data and structure refinement for [(C^N^C)Pt(PPh_3)].

Identification code	[(C^N^C)Pt(PPh_3)]
Empirical formula	$C_{35}H_{26}NPPt$
Formula weight	686.63
Temperature/K	99.94
Crystal system	monoclinic
Space group	$P2_1/c$
a/Å	13.9562(12)
b/Å	9.4262(8)
c/Å	20.5530(17)
α/°	90
β/°	95.744(6)
γ/°	90
Volume/Å^3	2690.3(4)
Z	4
ρ_{calc}g/cm^3	1.695
μ/mm^{-1}	5.300
F(000)	1344.0
Crystal size/mm^3	0.29 × 0.23 × 0.1
Radiation	MoKα (λ = 0.71073)
2Θ range for data collection/°	4.704 to 55.154
Index ranges	$-18 \leq h \leq 18$, $-12 \leq k \leq 12$, $-26 \leq l \leq 26$
Reflections collected	33575
Independent reflections	6210 [R_{int} = 0.1446, R_{sigma} = 0.1122]
Data/restraints/parameters	6210/0/343
Goodness-of-fit on F^2	0.915
Final R indexes [I>=2σ (I)]	R_1 = 0.0418, wR_2 = 0.0794
Final R indexes [all data]	R_1 = 0.0749, wR_2 = 0.0870
Largest diff. peak/hole / e Å^{-3}	1.50/-2.60

Crystal Data and Structural Refinement of [((C^N^C)Pt)$_2$(dppb)]

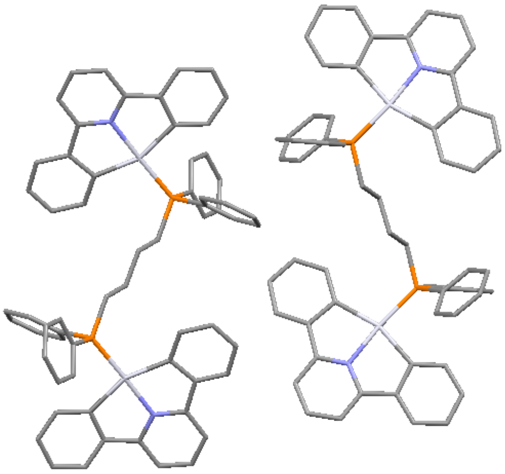

Table 3 Crystal data and structure refinement for [((C^N^C)Pt)$_2$(dppb)].

Identification code	[((C^N^C)Pt)$_2$(dppb)]
Empirical formula	$C_{62}H_{50}N_2P_2Pt_2$
Formula weight	1275.16
Temperature/K	105.83
Crystal system	monoclinic
Space group	P21/c
a/Å	17.0656(8)
b/Å	31.5329(17)
c/Å	9.2873(5)
α/°	90
β/°	104.798(2)
γ/°	90
Volume/Å3	4832.0(4)
Z	4
ρcalcg/cm3	1.753
μ/mm-1	5.894
F(000)	2488.0
Crystal size/mm3	0.54 × 0.43 × 0.39
Radiation	MoKα (λ = 0.71073)
2Θ range for data collection/°	3.574 to 53.928
Index ranges	-20 ≤ h ≤ 19, -37 ≤ k ≤ 36, -11 ≤ l ≤ 11
Reflections collected	65755
Independent reflections	8136 [Rint = 0.1112, Rsigma = 0.0653]
Data/restraints/parameters	8136/582/613
Goodness-of-fit on F2	0.968
Final R indexes [I>=2σ (I)]	R1 = 0.0407, wR2 = 0.0929
Final R indexes [all data]	R1 = 0.0573, wR2 = 0.0986
Largest diff. peak/hole / e Å-3	2.21/-2.72

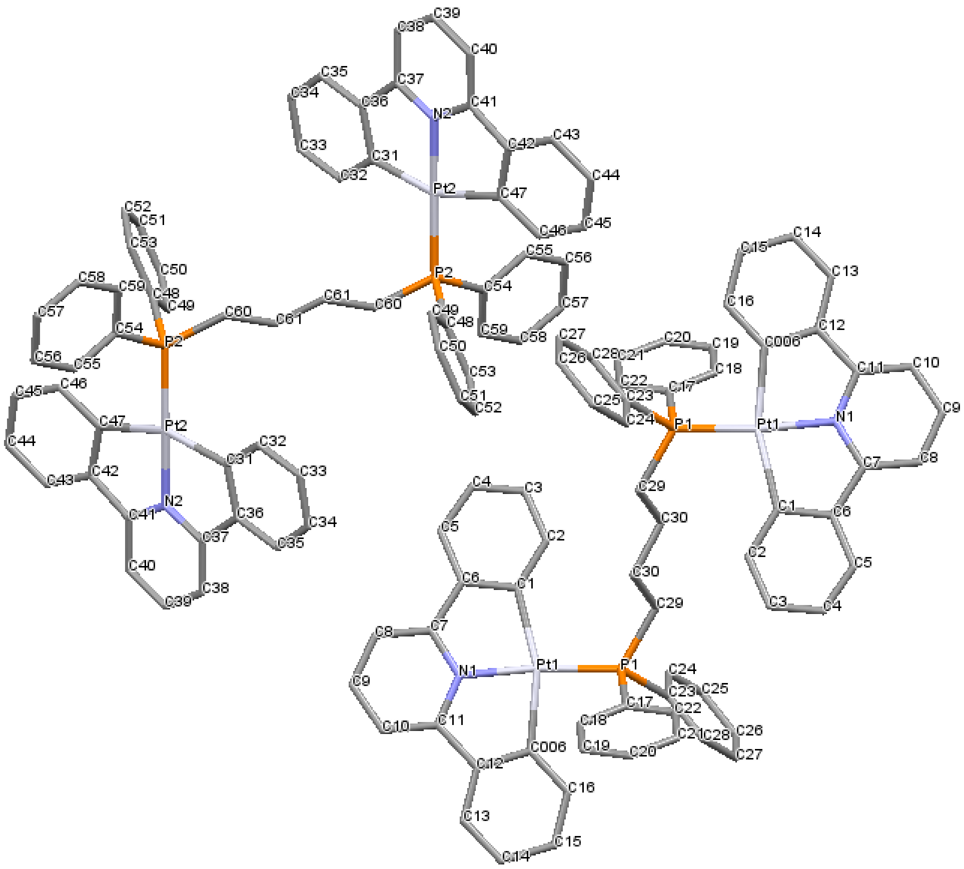

Table 4 Bond Lengths for [((C^N^C)Pt)$_2$(dppb)].

Atom	Atom	Length/Å	Atom	Atom	Length/Å
Pt2	P2	2.2190(17)	C49	C50	1.364(9)
Pt2	N2	2.025(5)	C54	C55	1.384(9)
Pt2	C47	2.091(6)	C54	C59	1.384(9)
Pt2	C31	2.085(7)	C41	C42	1.465(9)
Pt1	P1	2.2216(18)	C41	C40	1.398(9)
Pt1	C006	2.076(6)	C52	C51	1.379(10)
Pt1	N1	2.022(5)	C18	C19	1.396(9)
Pt1	C1	2.087(7)	C18	C17	1.384(9)
P1	C29	1.834(6)	C55	C56	1.397(9)
P1	C23	1.824(7)	C37	C36	1.476(9)
P1	C17	1.836(6)	C36	C31	1.407(9)
P2	C54	1.820(6)	C36	C35	1.389(9)
P2	C60	1.826(6)	C22	C17	1.396(9)
P2	C48	1.825(6)	C22	C21	1.375(9)
N2	C41	1.337(8)	C24	C25	1.405(9)
N2	C37	1.362(8)	C24	C23	1.384(9)
C006	C16	1.391(9)	C61	$C61^1$	1.529(12)
C006	C12	1.423(9)	C61	C60	1.535(8)
N1	C7	1.343(8)	C29	C30	1.525(8)
N1	C11	1.348(9)	C39	C40	1.371(9)

C16	C15	1.383(9)	C2	C3	1.382(10)
C45	C44	1.396(9)	C47	C42	1.415(9)
C45	C46	1.374(9)	C47	C46	1.404(9)
C15	C14	1.394(9)	C56	C57	1.394(9)
C12	C11	1.468(10)	C31	C32	1.396(9)
C12	C13	1.377(9)	C3	C4	1.380(10)
C38	C37	1.396(9)	C8	C9	1.372(10)
C38	C39	1.367(9)	C27	C26	1.390(9)
C7	C6	1.444(10)	C27	C28	1.395(9)
C7	C8	1.421(10)	C13	C14	1.389(10)
C58	C57	1.352(9)	C10	C9	1.382(10)
C58	C59	1.379(9)	C25	C26	1.373(9)
C6	C1	1.441(9)	C23	C28	1.388(9)
C6	C5	1.412(9)	C30	C30[2]	1.521(13)
C1	C2	1.393(10)	C19	C20	1.364(9)
C43	C44	1.379(9)	C5	C4	1.366(11)
C43	C42	1.403(9)	C34	C33	1.372(10)
C11	C10	1.392(9)	C34	C35	1.359(10)
C53	C52	1.379(9)	C33	C32	1.386(9)
C53	C48	1.405(9)	C20	C21	1.407(10)
C49	C48	1.392(9)	C51	C50	1.380(10)

[1]2-X,1-Y,1-Z; [2]1-X,1-Y,2-Z

Table 5 Bond Angles for [((C^N^C)Pt)$_2$(dppb)].

Atom	Atom	Atom	Angle/°	Atom	Atom	Atom	Angle/°
N2	Pt2	P2	176.31(14)	C40	C41	C42	127.3(6)
N2	Pt2	C47	79.5(2)	C53	C52	C51	120.0(7)
N2	Pt2	C31	80.0(2)	C17	C18	C19	120.2(6)
C47	Pt2	P2	97.98(18)	C54	C55	C56	120.7(6)
C31	Pt2	P2	102.71(18)	N2	C37	C38	118.0(6)
C31	Pt2	C47	158.9(3)	N2	C37	C36	113.0(5)
C006	Pt1	P1	94.41(18)	C38	C37	C36	129.0(6)
C006	Pt1	C1	159.8(3)	C31	C36	C37	116.8(6)
N1	Pt1	P1	171.52(15)	C35	C36	C37	120.3(6)
N1	Pt1	C006	80.0(2)	C35	C36	C31	122.9(6)
N1	Pt1	C1	80.2(3)	C21	C22	C17	121.2(6)
C1	Pt1	P1	105.7(2)	C23	C24	C25	121.1(6)
C29	P1	Pt1	121.7(2)	C61[1]	C61	C60	110.6(6)
C29	P1	C17	101.1(3)	C30	C29	P1	115.7(4)
C23	P1	Pt1	113.3(2)	C61	C60	P2	113.7(4)
C23	P1	C29	98.7(3)	C38	C39	C40	121.3(6)
C23	P1	C17	109.0(3)	C3	C2	C1	123.9(7)
C17	P1	Pt1	111.5(2)	C42	C47	Pt2	112.0(4)
C54	P2	Pt2	113.0(2)	C46	C47	Pt2	132.7(5)
C54	P2	C60	102.1(3)	C46	C47	C42	114.9(6)

C54	P2	C48	106.5(3)	C57	C56	C55	118.9(6)
C60	P2	Pt2	120.8(2)	C36	C31	Pt2	112.1(5)
C48	P2	Pt2	113.9(2)	C32	C31	Pt2	133.5(5)
C48	P2	C60	98.6(3)	C32	C31	C36	114.5(6)
C41	N2	Pt2	118.3(4)	C4	C3	C2	120.4(8)
C41	N2	C37	123.7(6)	C9	C8	C7	119.1(7)
C37	N2	Pt2	118.0(4)	C26	C27	C28	120.5(6)
C16	C006	Pt1	132.6(5)	C43	C42	C41	121.2(6)
C16	C006	C12	115.6(6)	C43	C42	C47	122.5(6)
C12	C006	Pt1	111.8(5)	C47	C42	C41	116.1(6)
C7	N1	Pt1	117.6(5)	C58	C57	C56	120.0(6)
C7	N1	C11	124.0(6)	C12	C13	C14	121.0(7)
C11	N1	Pt1	118.1(4)	C9	C10	C11	119.0(7)
C15	C16	C006	122.7(7)	C58	C59	C54	120.2(6)
C46	C45	C44	120.4(6)	C39	C40	C41	119.0(6)
C16	C15	C14	120.7(7)	C26	C25	C24	119.8(6)
C006	C12	C11	116.3(6)	C24	C23	P1	116.1(5)
C13	C12	C006	121.9(6)	C24	C23	C28	118.7(6)
C13	C12	C11	121.8(6)	C28	C23	P1	125.2(5)
C39	C38	C37	119.3(6)	C45	C46	C47	123.2(7)
N1	C7	C6	114.7(6)	C25	C26	C27	119.6(6)
N1	C7	C8	117.8(7)	C30[2]	C30	C29	111.7(7)
C8	C7	C6	127.4(6)	C53	C48	P2	122.0(5)
C57	C58	C59	121.3(7)	C49	C48	P2	119.6(5)
C1	C6	C7	116.2(6)	C49	C48	C53	118.3(6)
C5	C6	C7	122.8(6)	C20	C19	C18	120.7(7)
C5	C6	C1	121.0(7)	C18	C17	P1	119.0(5)
C6	C1	Pt1	110.9(5)	C18	C17	C22	118.7(6)
C2	C1	Pt1	134.2(5)	C22	C17	P1	122.1(5)
C2	C1	C6	114.5(6)	C4	C5	C6	120.9(7)
C44	C43	C42	119.9(6)	C35	C34	C33	120.0(7)
N1	C11	C12	113.3(6)	C13	C14	C15	118.1(7)
N1	C11	C10	119.0(7)	C23	C28	C27	120.4(6)
C10	C11	C12	127.7(7)	C34	C33	C32	119.9(7)
C43	C44	C45	119.0(6)	C5	C4	C3	119.3(7)
C52	C53	C48	120.3(6)	C19	C20	C21	119.6(7)
C50	C49	C48	120.9(7)	C22	C21	C20	119.5(6)
C55	C54	P2	118.1(5)	C52	C51	C50	120.1(7)
C59	C54	P2	123.0(5)	C49	C50	C51	120.4(7)
C59	C54	C55	118.9(6)	C8	C9	C10	120.9(7)
N2	C41	C42	114.1(6)	C33	C32	C31	122.9(7)
N2	C41	C40	118.6(6)	C34	C35	C36	119.8(7)

[1]2-X,1-Y,1-Z; [2]1-X,1-Y,2-Z

Solid-State Analysis of [((CNC)Pt)$_2$(dppb)]

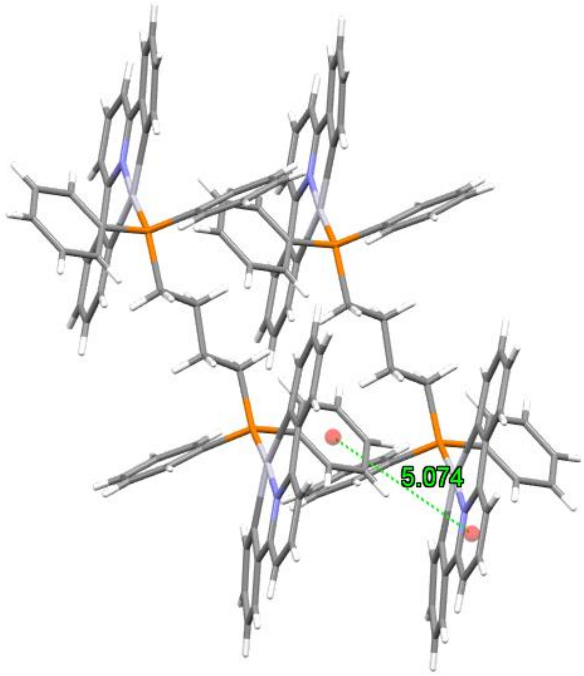

Figure S1. Solid-state analysis of [((C^N^C)Pt)$_2$(dppb)] highlighting the edge-to-face π-π stacking interactions present.[7,8]

Chemosensitivity Studies

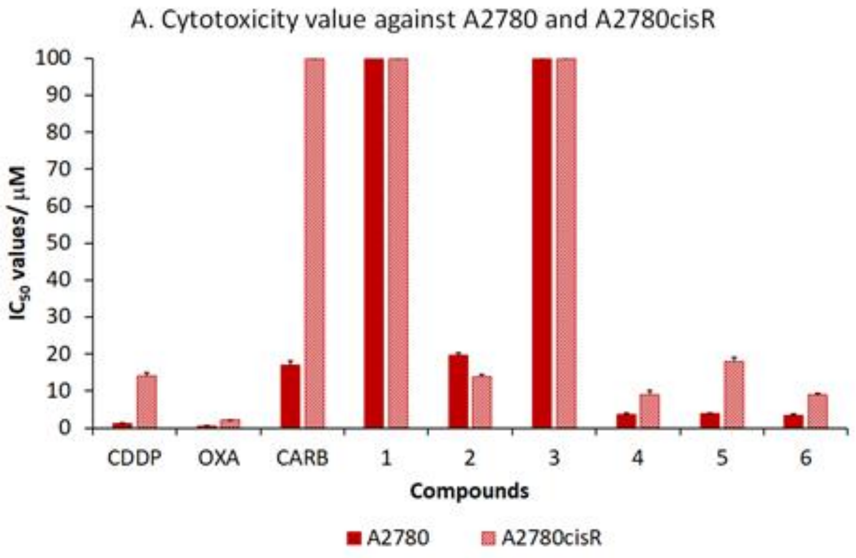

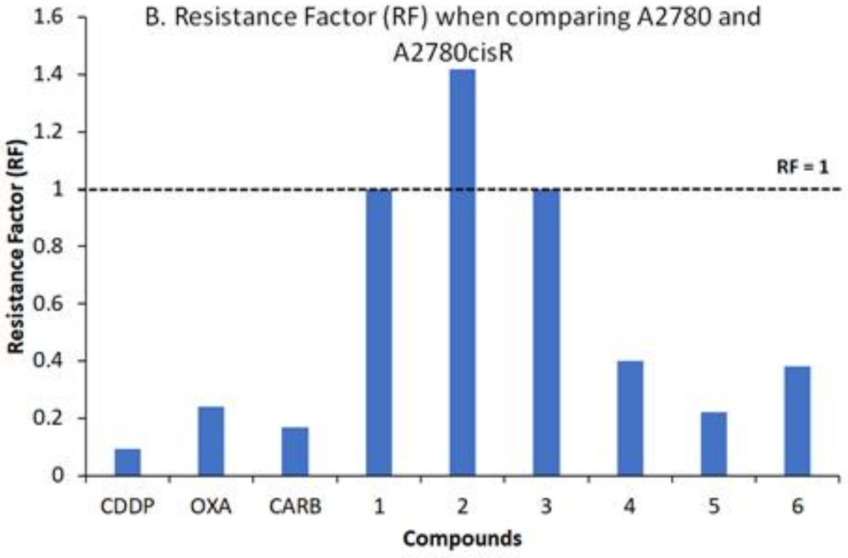

Figure S2. a) IC_{50} values against A2780, A2780cisR, and **b)** Resistance Factor (RF) for ligand **1** and complexes **2-6**, cisplatin (**CDDP**), carboplatin (**CARB**) and oxaliplatin (**OXA**). RF < 1 indicates selectivity for A2780, RF = 1 indicates equitoxicity and RF > 1 indicates selectivity for the cisplatin-resistant ovarian carcinoma cell line, A2780cisR.

Appendix: NMR spectra

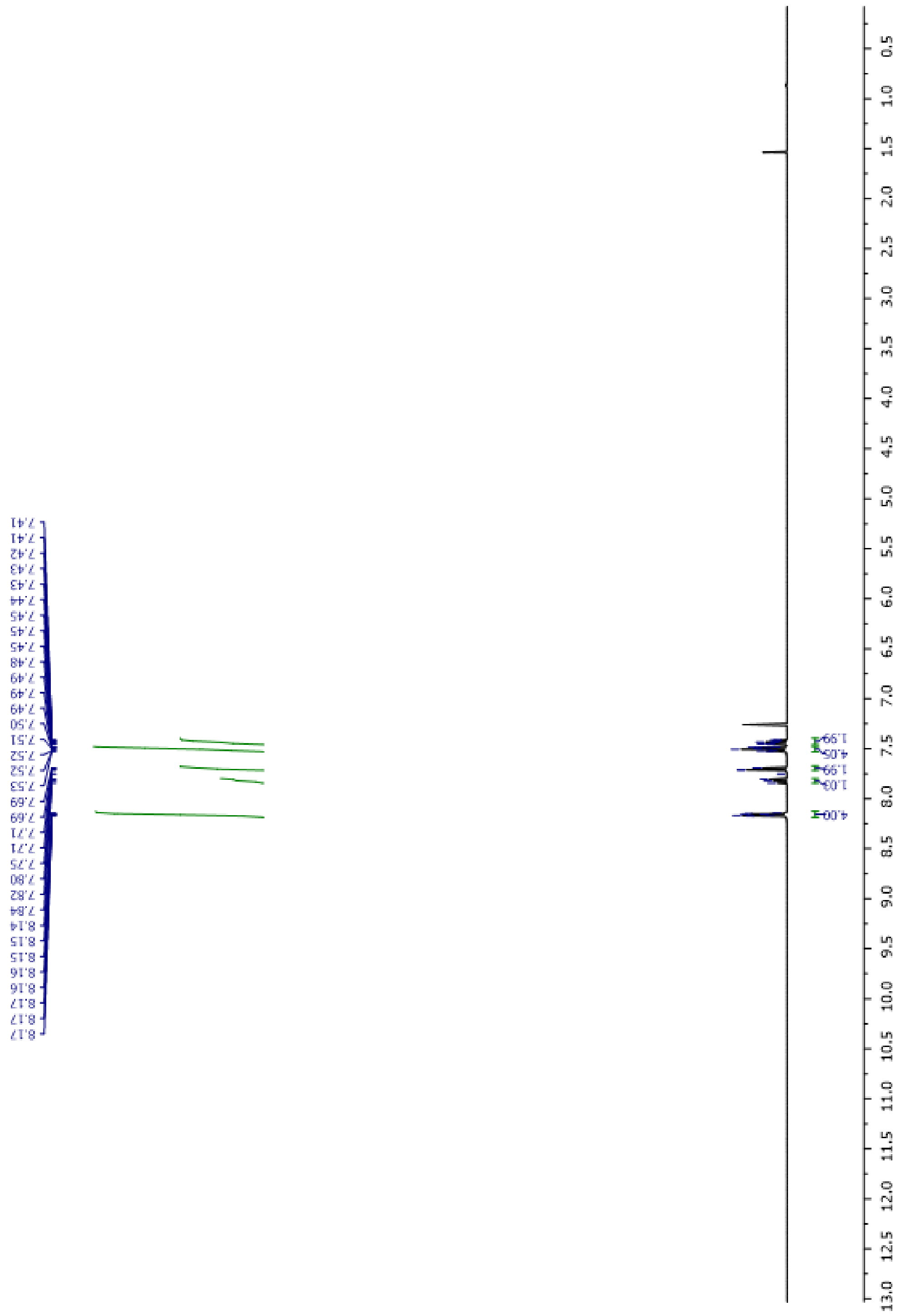

^1H NMR (400 MHz, $CDCl_3$, 298 K) spectrum of H_2L

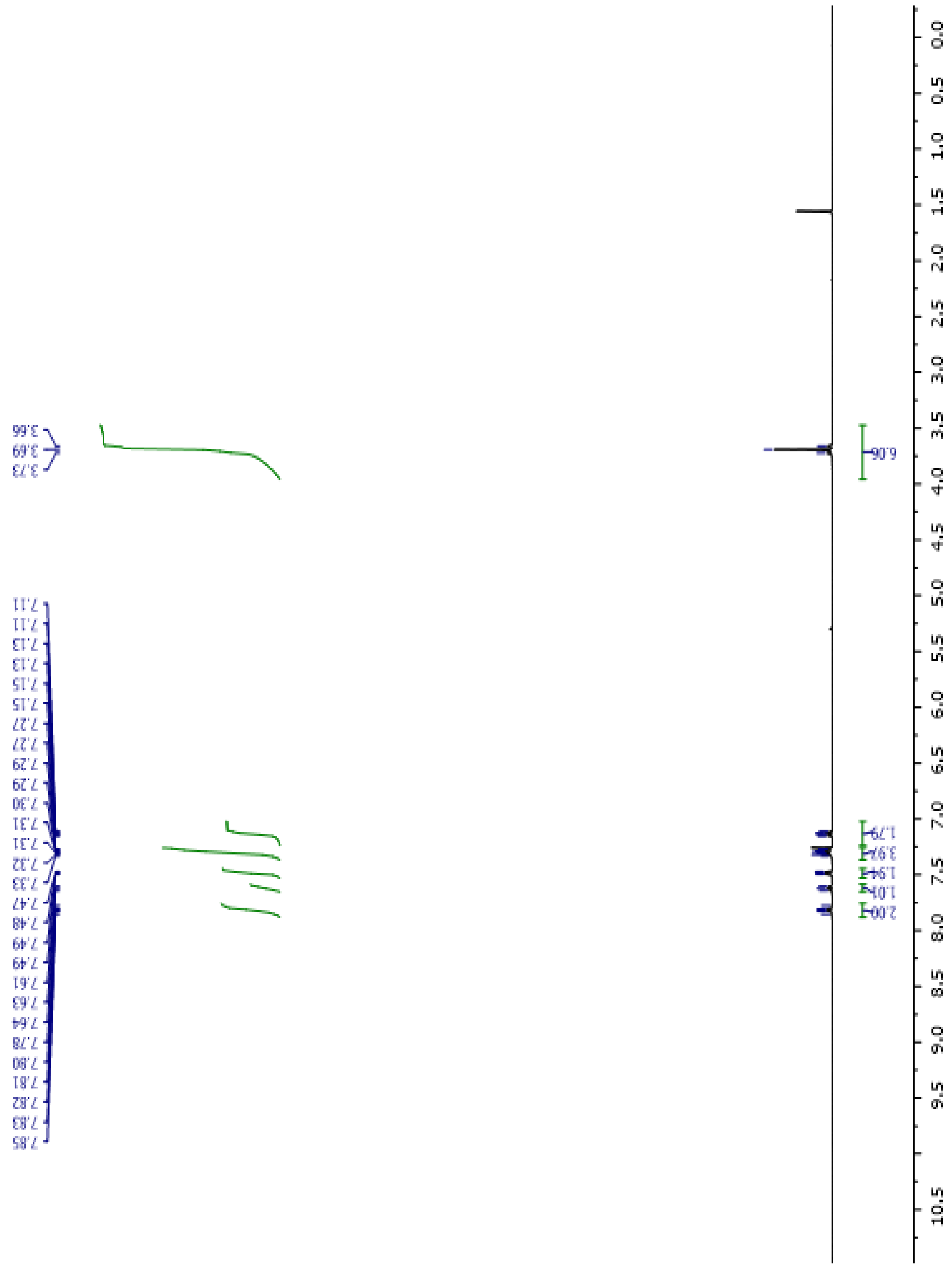

^{1}H NMR (400 MHz, $CDCl_3$, 298 K) spectrum of [(C^N^C)Pt(DMSO)]

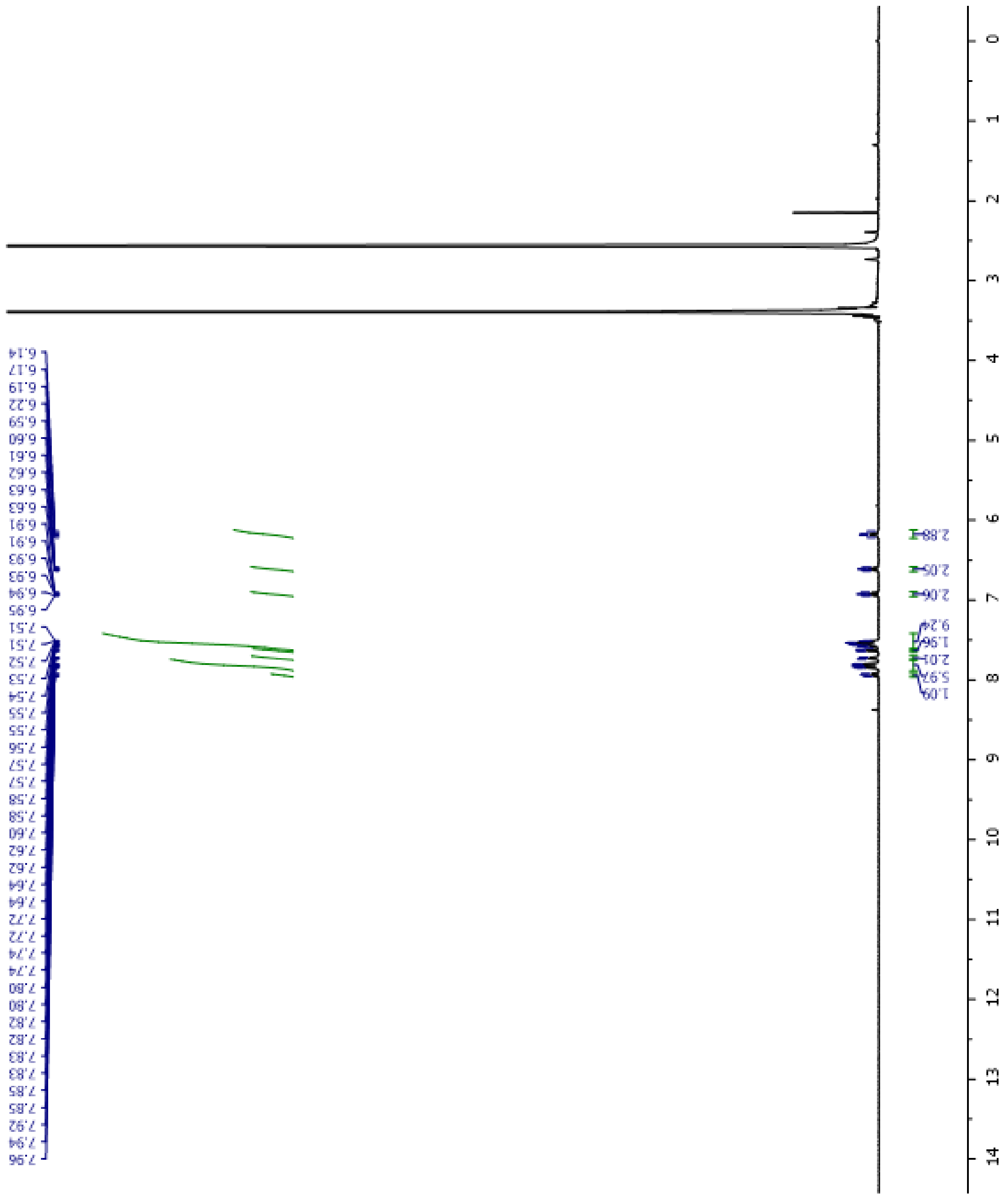

^{1}H NMR (400 MHz, $CDCl_3$, 298 K) spectrum of [(C^N^C)Pt(PPh_3)]

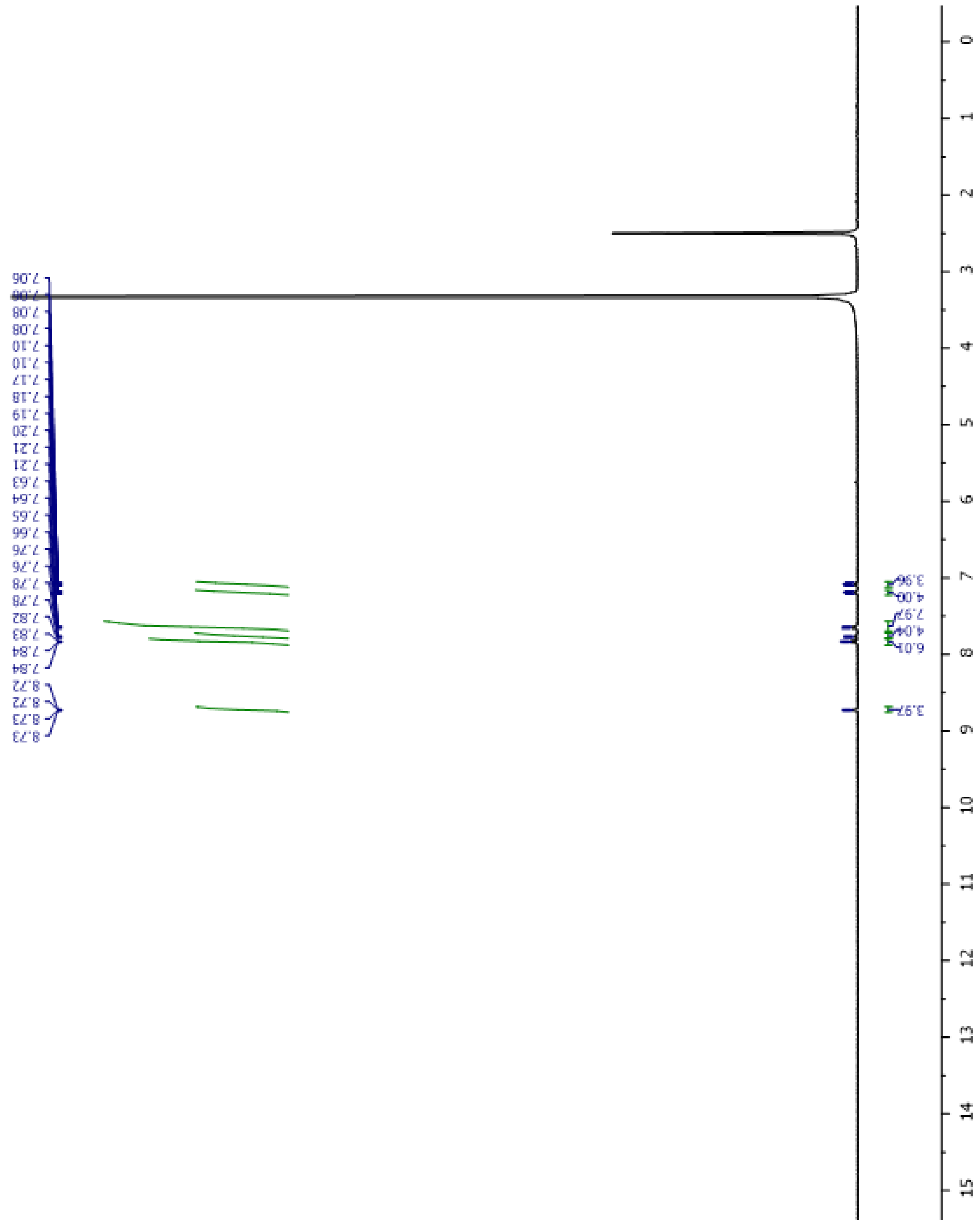

^{1}H NMR (400 MHz, DMSO-d_6, 298 K) spectrum of [((C^N^C)Pt)$_2$(4,4`-bipy)]

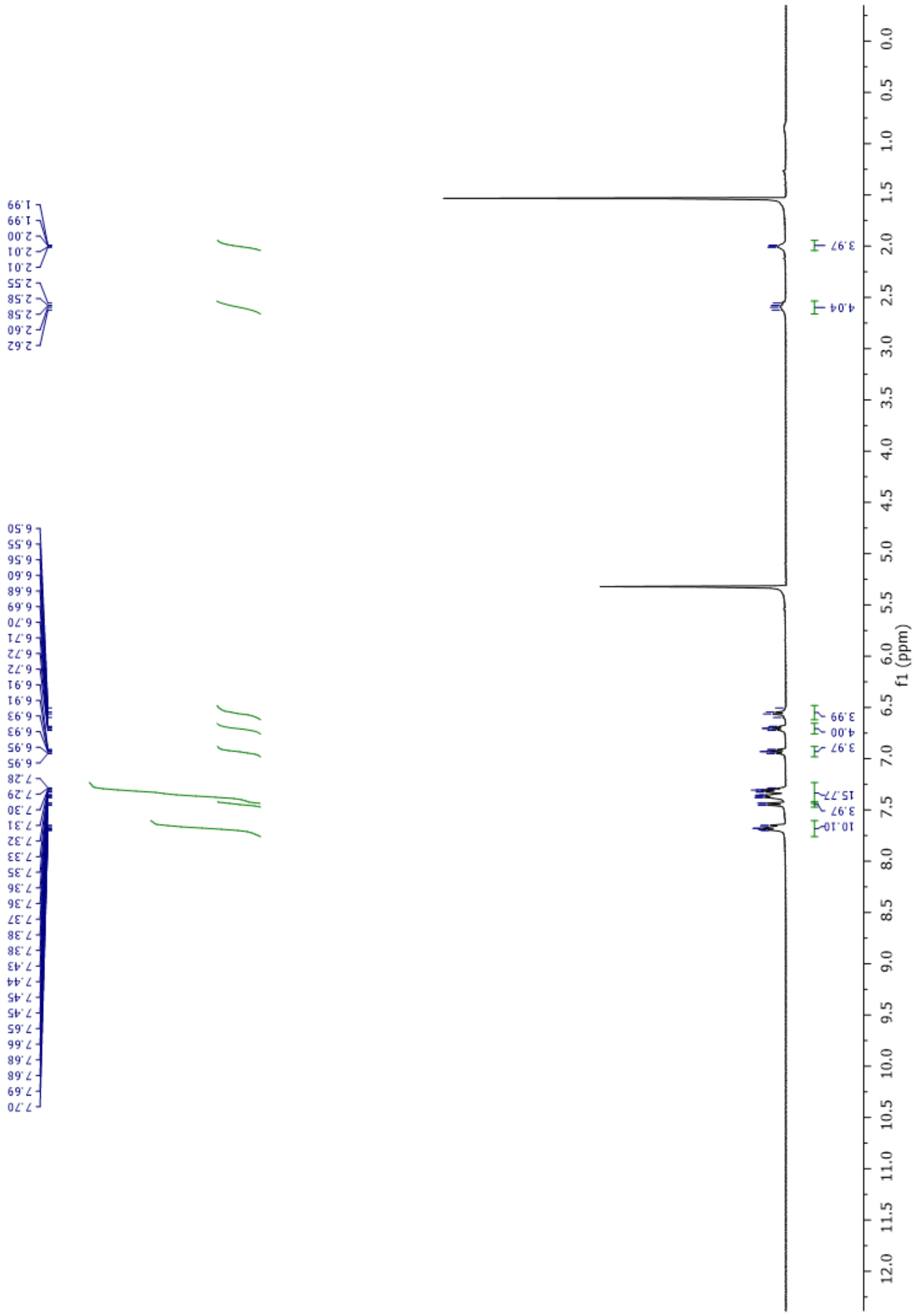

^{1}H NMR (400 MHz, CD_2Cl_2, 298 K) spectrum of [((C^N^C)Pt)$_2$(dppb)]

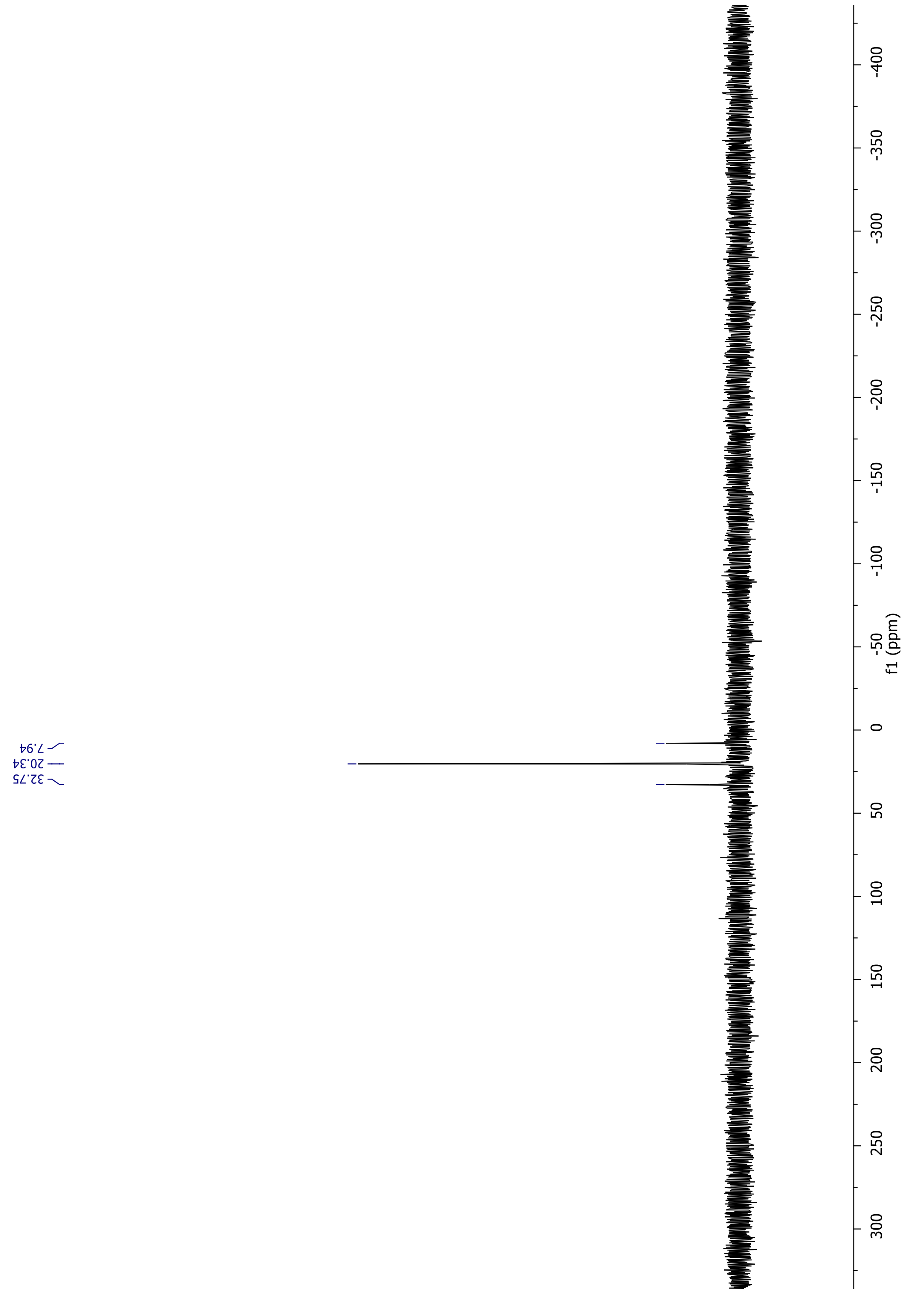

$^{31}P\{^1H\}$ NMR (162 MHz, CD_2Cl_2, 298 K) spectrum of [((C^N^C)Pt)$_2$(dppb)]

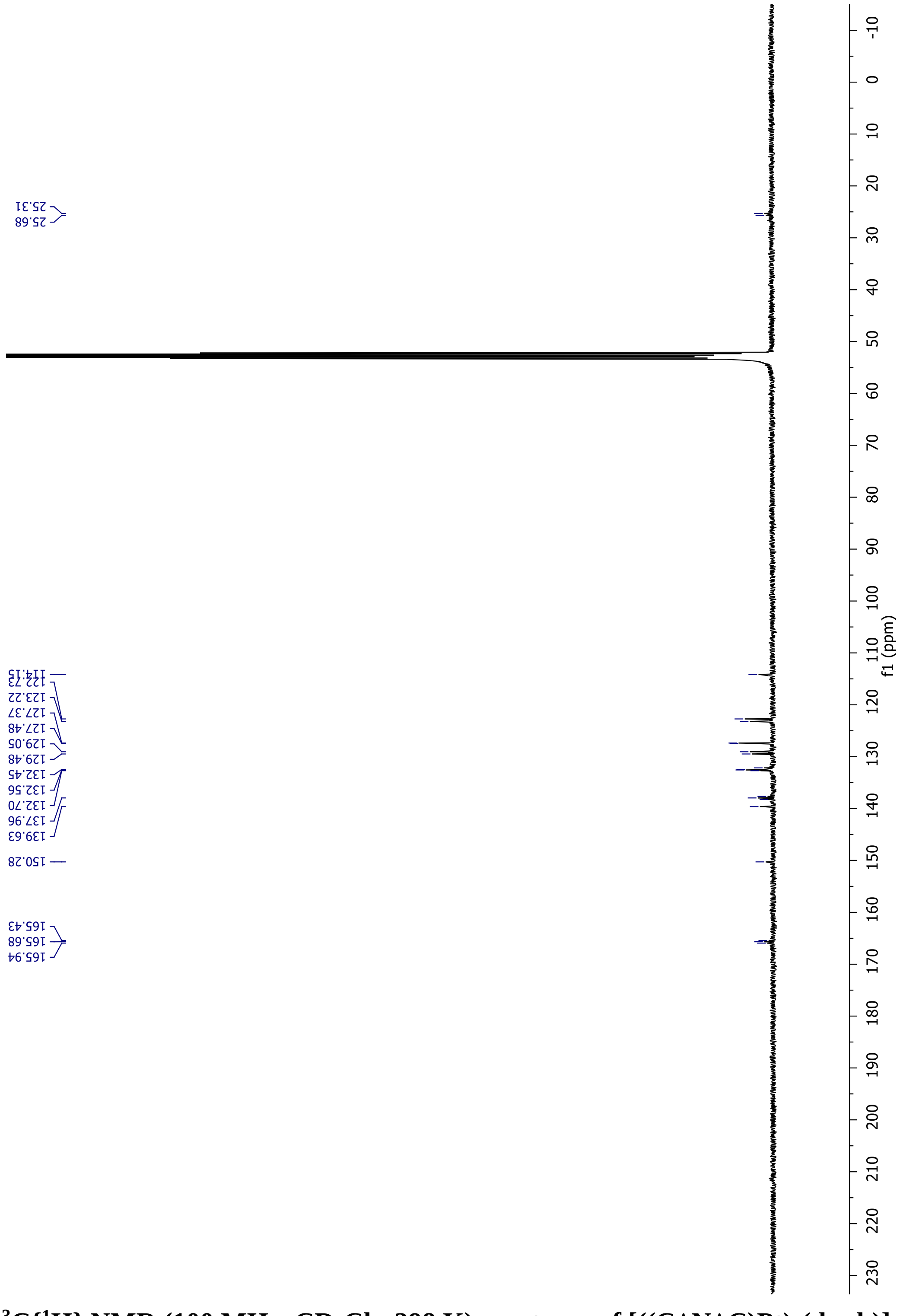

$^{13}C\{^{1}H\}$ NMR (100 MHz, CD_2Cl_2, 298 K) spectrum of [((C^N^C)Pt)$_2$(dppb)]

References

1. G. W. V. Cave, N. W. Alcock, J. P. Rourke, *Organometallics* **1999**, *18*, 1801.

2. M. Baya, U. Belío, I. Fernández, S. Fuertes, A. Martín, *Angew. Chem. Int. Ed.* **2016**, *55*, 6978-6982.

3. G. M. Sheldrick, *Acta Crystallogr. Sect. A: Crystallogr.*, **2015**, *71*, 3.

4. G. M. Sheldrick, *Acta Crystallogr. Sect. A Crystallogr.*, **2008**, *64*, 112.

5. O. V. Dolomanov, L. J. Bourhis, R. J. Gildea, J. A. K. Howard and H. Puschmann, *J. Appl. Cryst.*, **2009**, *42*, 339.

6. (a) C. F. Macrae, I. J. Bruno, J. A. Chisholm, P. R. Edgington, P. McCabe, E. Pidcock, L. Rodriguez-Monge, R. Taylor, J. van de Streek and P. A. Wood, *J. Appl. Cryst.*, **2008**, *41*, 466; (b) C. F. Macrae, P. R. Edgington, P. McCabe, E. Pidcock, G. P. Shields, R. Taylor, M. Towler and J. van de Streek, *J. Appl. Cryst.*, **2006**, *39*, 453.

7. C. Hunter, J. Singh, J. Thornton, *J. Chem. Soc. Perkin Trans. 2*, **2001**, 651.

8. E. R. T. Tierink, J. Zuckerman-Schpector, The Importance of Pi-Interactions in Crystal Engineering, Frontiers in Crystal Engineering, *Wiley*, 2012.
